# CU Cilia – an application for image analysis by machine learning – reveals significance of cysteine cathepsin K activity for primary cilia of human thyroid epithelial cells

**DOI:** 10.3389/fendo.2025.1588394

**Published:** 2025-11-27

**Authors:** Maren Rehders, Kseniia Alekseitseva, João Gissoni, Alara G. Doğru, Petr Popov, Andrei Boiarov, Klaudia Brix

**Affiliations:** 1 School of Science, Constructor University, Bremen, Germany; 2 Constructor Technology AG, Schaffhausen, Switzerland; 3 Constructor Knowledge Labs, Bremen, Germany

**Keywords:** cysteine cathepsins, image analysis, machine learning, neural network pre-training, odanacatib, primary cilia, protease inhibitors, thyroid epithelial cells

## Abstract

**Introduction:**

Cysteine cathepsins K and L are crucial for proper thyroid function in mice. Inhibition of cysteine peptidases in rodent thyroid epithelial cells *in vitro* results in loss of primary cilia, highlighting the significance of proteolysis for cilia maintenance.

**Methods:**

In this study, we used an *in vitro*-approach to ask whether primary cilia of the normal human thyroid epithelial cell line Nthy-ori 3–1 are affected by cysteine cathepsin inhibition in numbers and/or structure. To interpret hundreds of primary cilia in dozens of high-resolution laser-scanning microscopic images, an objective image analysis approach is essential.

**Results:**

Therefore, we developed CU Cilia, *i.e.*, a method for the detection of primary cilia and for segmentation of nuclei by machine learning-based image analysis algorithms. For validation of CU Cilia, the results of nuclei segmentation and primary cilia detection are compared with the image analysis software Cell Profiler™. While manual editing by an image analysis expert enhances the already very good image segmentation results when using Cell Profiler™, the herein described CU Cilia application achieved well-comparable results. CU Cilia even allows non-expert users to perform image analysis with little to no training. CU Cilia uses machine learning by a U-Net neural network simplified architecture and requires only a few images as training datasets. Both approaches, Cell Profiler™ and the CU Cilia application, revealed that the broad-spectrum cysteine peptidase inhibitor E64d and the specific cysteine cathepsin K inhibitor Odanacatib affect primary cilia structure significantly, while their numbers remained unaffected.

**Discussion:**

The results point to an elongation of primary cilia in the Odanacatib-treated Nthy-ori 3–1 thyroid epithelial cells, supporting the notion that cysteine cathepsin K activity is physiologically important not only in murine but also in human thyroid cells.

## Graphical Abstract

**Figure:**
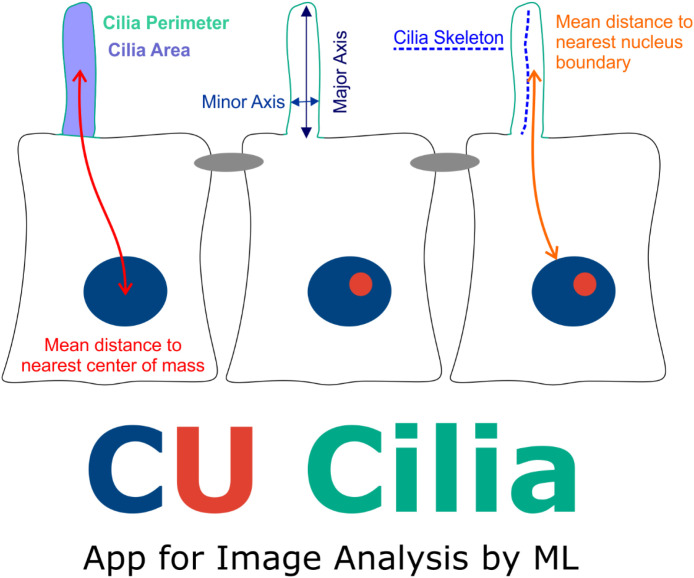

## 1 Introduction

Primary cilia are non-motile cellular appendages of most eukaryotic cells with a distinct composition of their cytoplasmic and plasma membrane constituents (1, 2). The molecules uniquely present in and at primary cilia enable tasks in chemo-, mechano- and temperature sensing of the peri-cellular environment (3–5). Thus, primary cilia function as cellular antennas and bear critical roles in signaling during development, in physiology and in disease (6–8). Disturbances in the genetic make-up of primary ciliary molecules are the underlying causes of ciliopathies (9). Hence, in current understanding, changes in their molecular composition, typically resulting in altered frequencies and morphologies of primary cilia, are considered both causative and indicative of a broad variety of diseases (10, 11).

The primary cilia of the thyroid gland are less well characterized in comparison to those of, *e.g.*, the kidney, but their importance for the maintenance of fully differentiated states of thyroid epithelial cells is no longer in question (2, 8, 12–14). In thyroid pathology, it is first and foremost thyroid cancer which is characterized by a loss of primary cilia from dedifferentiated thyroid carcinoma cells (8). Because shortened or lacking primary cilia are equivalent to altered signaling events, it is proposed that cell cycle disturbances or mitochondria-dependent apoptosis are early events in tumorigenesis of the thyroid (8, 15, 16). Very recently, several original papers and a comprehensive review article further highlighted and summarized the observations regarding alterations of primary cilia in common thyroid disorders like autoimmune thyroid disease (17, 18), hypothyroidism and thyroid nodules (19) or even in rare inherited conditions like Pendred syndrome (20). In summary, it is critically important to monitor primary cilia features as determinators of thyroid health and as indicators of thyroid disease severity. Thus, visualizing primary cilia and determining their frequencies and structural features by image analysis is essential for investigations on thyroid physiology and pathology.

With the advances of improved microscopic imaging, a wealth of high-content and high-quality images taken from cells or tissues await critical interpretation. Hence, deep learning-based computer vision methods provide powerful tools to characterize, *e.g.*, primary cilia. Typically, such tools comprise neural networks that solve segmentation problems and are trained on large datasets of images (21). To improve performance of such models, one can search for optimal parameters of the model or, alternatively, fine-tune the method by providing labeled datasets of new images. However, both options require an expert, either trained to use a computer vision method or a data scientist, as well as substantial time.

In this study we have set out to develop an intuitive and easy-to-apply tool for the detection and description of primary cilia in immuno-stained cells. Inspired by numerous cilia detection tools of various complexity, we herein describe the construction of an App that uses machine learning (ML)-based image analysis algorithms. The App is called “CU Cilia”, and it has been thoroughly assessed and evaluated by comparing the processing times and results with those achieved from the same datasets analyzed by CellProfiler™ (RRID: SCR_007358) (22) and Cellpose (RRID: SCR_021716) (21, 23).

Other cilia-describing tools, such as ACDC (24), CiliaQ (25), detectCilia (26), or the Cilialyzer (27) were not included in comparative testing modes, due to their complexity, time-consuming user-training and fine-tuning of the software settings. Rationalized deep learning was developed recently for lattice light sheet microscopy specifically to image motile cilia in living cells (28). This truly most elegant approach is not thought to image cilia upon immunostaining, which is however the standard technique for visualization of the cellular antennas by cell biologists or pathologists.

The result of our evaluation is the introduction of CU Cilia, which is freely accessible after signing up or logging into the Constructor Platform (https://constructor.app). This tool provides a powerful resource for basic researchers and clinicians without a thorough background in data science, to determine the frequency, eccentricity, lengths, and other parameters of primary cilia in immuno-stained cells.

Cysteine cathepsins B, K, and L are important for thyroid function in mice (29). Inhibition of cysteine cathepsins B and L in rat thyrocytes resulted in cilia loss (30), while inhibition of all cysteine peptidases by E64 and/or DCG-04 in human Nthy-ori 3–1 cells resulted in cilia shortening (31). Therefore, we were interested in using similar pharmacological interventions in this study. We now use specific inhibitors of the cysteine cathepsins B, K, and L and compared the results with those using E64d as a broad-spectrum inhibitor. We show that elongation of primary cilia results from inhibition of cysteine peptidase activities by E64d, and specifically of cathepsin K by Odanacatib treatment of cultured Nthy-ori 3–1 cells.

We conclude that studies on primary cilia as biomarkers of health and disease as well as upon pharmacological intervention will be facilitated and promoted by CU Cilia-based image analysis.

## 2 Materials and methods

### 2.1 Cell culture

Normal human thyroid follicular epithelial cells (Nthy-ori 3–1 cell line; Sigma Aldrich, Taufkirchen, Germany, #90011609, RRID: CVCL_2659) were cultured in Roswell Park Memorial Institute medium (#RPMI-A, Capricorn Scientific, Hessen, Germany) containing 10% fetal calf serum (#FBS-11A, Capricorn Scientific, Hessen, Germany). Cell cultures were maintained at 37°C in a humidified atmosphere at 5% CO_2_. Prior to cysteine peptidase inhibition, Nthy-ori 3–1 cells were grown in serum-free RPMI for 24–48 h to induce cell cycle arrest at the G_1_-S transition (32), thereby promoting formation of primary cilia (33).

### 2.2 Protease inhibition experiments

Cysteine peptidase activities were inhibited in Nthy-ori 3–1 cell cultures as previously described (31). However, in this study, cell cultures were incubated for 1 h and 24 h with 10 µM E64d (#BML-PI107, Enzo Life Sciences, Lörrach, Germany) for broad-spectrum inhibition of cysteine peptidase activities and with cysteine cathepsin B-, K-, and L-specific inhibitors, namely, 10 µM CA074 me (#BML-PI126, Enzo Life Sciences, Lörrach, Germany), 10 µM Odanacatib (#S1115, Selleck Chemicals GmbH, Cologne, Germany), and 10 µM cathepsin L inhibitor III (#219427, Merck KGaA, Darmstadt, Germany), respectively. As a solvent control, 0.2% DMSO (#4720.1, Carl Roth GmbH, Karlsruhe, Germany) was used. Visualization of proteolytically active cysteine peptidases was by means of activity-based probes (ABPs), for which Nthy-ori 3–1 cells were incubated for 1 h with 1 µM DCG-04 Green (34) that binds irreversibly to the active site of cysteine peptidases in a 1:1 molar fashion, thus, also acts as a broad-spectrum inhibitor of cysteine peptidases (31, 35).

### 2.3 Immunostaining

In all immunofluorescence experiments, Nthy-ori 3–1 cells were fixed with 4% paraformaldehyde (PFA) in 200 mM HEPES at pH 7.4 for 15 min at room temperature and permeabilized for 5 min with ice-cold methanol (#4627.5, Carl Roth GmbH, Karlsruhe, Germany). The cells were washed with calcium- and magnesium-free PBS (CMF-PBS), which consisted of 1.5 mM NaH_2_PO_4_, 8.1 mM Na_2_HPO_4_, 2.7 mM KCl, and 0.15 mM NaCl, for 15 min before blocking of non-specific binding sites with 3% BSA (#11930.04, Bovine Serum Albumin, Serva Electrophoresis GmbH, Heidelberg, Germany) in CMF-PBS at 37°C for 1 h. Cells were then washed with 0.1% BSA in CMF-PBS. For primary cilia detection, rabbit anti-human/mouse/rat/dog ARL13B (#17711-1-AP, 1:250, Proteintech, Planegg, Germany, RRID: AB_2060867) was used, followed by secondary antibody incubation, namely, goat anti-rabbit Alexa Fluor 546 or Alexa Fluor plus 488 F(ab’) fragments (1:200, Thermo Fisher Scientific, Bremen, Germany, #A11071, RRID: AB_2534115, and #A48282TR, RRID: AB_2896346, respectively) were used. The following primary antibodies were used for cysteine cathepsin immunostaining, *i.e.*, goat anti-mouse cathepsin B (1:50, Neuromics *via* Acris Antibodies GmbH, Herford, Germany, #GT15047, RRID: AB_1611799), mouse anti-human cathepsin K (1:10, Merck KGaA, Darmstadt, Germany, #IM55, RRID: AB_877796), and goat anti-mouse cathepsin L (1:50, Neuromics *via* Acris Antibodies GmbH, Herford, Germany, #GT15049, RRID: AB_1611804). Secondary antibodies were Alexa Fluor 546-conjugated donkey anti-goat (1:200, Thermo Fisher Scientific, Bremen, Germany, #A-11056, RRID: AB_2534103), Alexa Fluor 405 conjugated goat anti-mouse (1:200, Thermo Fisher Scientific, Bremen, Germany, #A48255, RRID:_AB2890536) and Alexa Fluor 488 conjugated goat anti-rabbit IgG (1:200, Thermo Fisher Scientific, Bremen, Germany, #A-11070, RRID: AB_2534114). All secondary antibodies were diluted 1:200 in 0.1% BSA in CMF-PBS. To counterstain nuclear DNA, Draq5™ (Bio Status Limited, Shepshed Leicestershire, UK, RRID: AB_2869620) was used at a final concentration of 5 µM. Immuno-stained cells grown on coverslips were rinsed in ddH_2_O and mounted in Mowiol (14% Mowiol 4-88, Carl Roth GmbH, Karlsruhe, Germany, #0713, in 33% glycerol).

### 2.4 Image acquisition

Immuno-stained specimens were imaged with a confocal laser scanning microscope (LSM) equipped with Diode lasers and Diode Pumped Solid State (DPSS) lasers (LSM 980, Carl Zeiss Jena GmbH, Jena, Germany). Micrographs were obtained at resolutions of 300 dpi, in 16- or 24-bit scanning-modes, and at sizes of 2024 x 2024 and 3238 x 3226 pixels, taken with the LSM 980 ZEISS ZEN Microscopy software, release 3.4 (Carl Zeiss Jena GmbH, Jena, Germany, RRID: SCR_013672), stored in CZI-format and exported to TIFF-format using the Zeiss Zen software, release 3.4. Z-stacks were taken from differently treated Nthy-ori 3–1 cells at 7–9 focal planes with 1.00 µm distances, respectively, and were displayed and analyzed as extended focus (EF) TIFF-images.

### 2.5 Image analysis with CellProfiler™

Standard input images must meet the following requirements: namely, pixel sizes between 2024 x 2024 and 3168 x 3168, RGB color mode, a resolution of 300 dpi, depth of 16 or 24 bit, and TIFF-file format. Analysis of primary cilia was done using the open-source automated image analysis software CellProfiler™ Image Analysis Software (release 4.2.6, RRID: SCR_007358) (22). To quantify cilia frequencies and lengths, two different pipelines, independently created by two different users (CellProfiler™ “expert” and CellProfiler™ “non-expert”) with different module arrangements were used. The pipelines with detailed settings are denoted “expert” and “non-expert” and can be found in the **Supplementary Tables 1**, **2**.

#### 2.5.1 CellProfiler™ pipeline “expert”

The module “ColorToGray” splits the input image into respective channels, *i.e.*, green, red, and blue. “RescaleIntensity” reduces the background intensity for the green (cilia) channel. The “EnhanceOrSuppressFeatures” module was used for the green (cilia) channel to improve subsequent identification of cilia. The “IdentifyPrimaryObjects” module aims to identify the total number of nuclei and cilia, respectively. “MeasureObjectSizeShape” allows determination of cilia architecture including parameters like minor/major axis length, area, form factor, eccentricity, perimeter, etc. “FilterObjects” was used to exclude false positively identified cilia. To visualize identified primary cilia or nuclei, “OverlayOutlines” and “Display-DataOnImage” were included. For data analysis and image curation, the modules “SaveImages and ExportToSpreadsheet” were used. The diameters of nuclei were restricted to range from 180–750 in pixel units, to exclude apoptotic bodies, and the diameters of cilia were restricted to the range of 18–220 in pixel units, to exclude mere staining of forming cilia at the MTOC (microtubule-organizing center). Nuclei and primary cilia touching the borders of an image were excluded from the analyses. The module “EditObjectsManually” was used for a subset of 6 arbitrarily chosen images that served as “ground truth” information for cilia lengths determinations by CU Cilia and for benchmarking of CellProfiler™ *versus* CU Cilia acquired data.

#### 2.5.2 CellProfiler™ pipeline “non-expert”

The non-expert pipeline consisted of the modules as described above, namely, “ColorToGray”, “IdentifyPrimaryObjects”, “MeasureObjectSizeShape”, “OverlayOutlines”, “Display-DataOnImage”, “SaveImages and ExportToSpreadsheet” were used. Nuclei diameters ranged from 245–500 in pixel units, and the cilia lengths were in the range of 15–90 in pixel units. Primary cilia touching the image borders, but not nuclei, were excluded from the analyses.

### 2.6 Building the CU Cilia nuclei segmentation model

**Figure 1:**
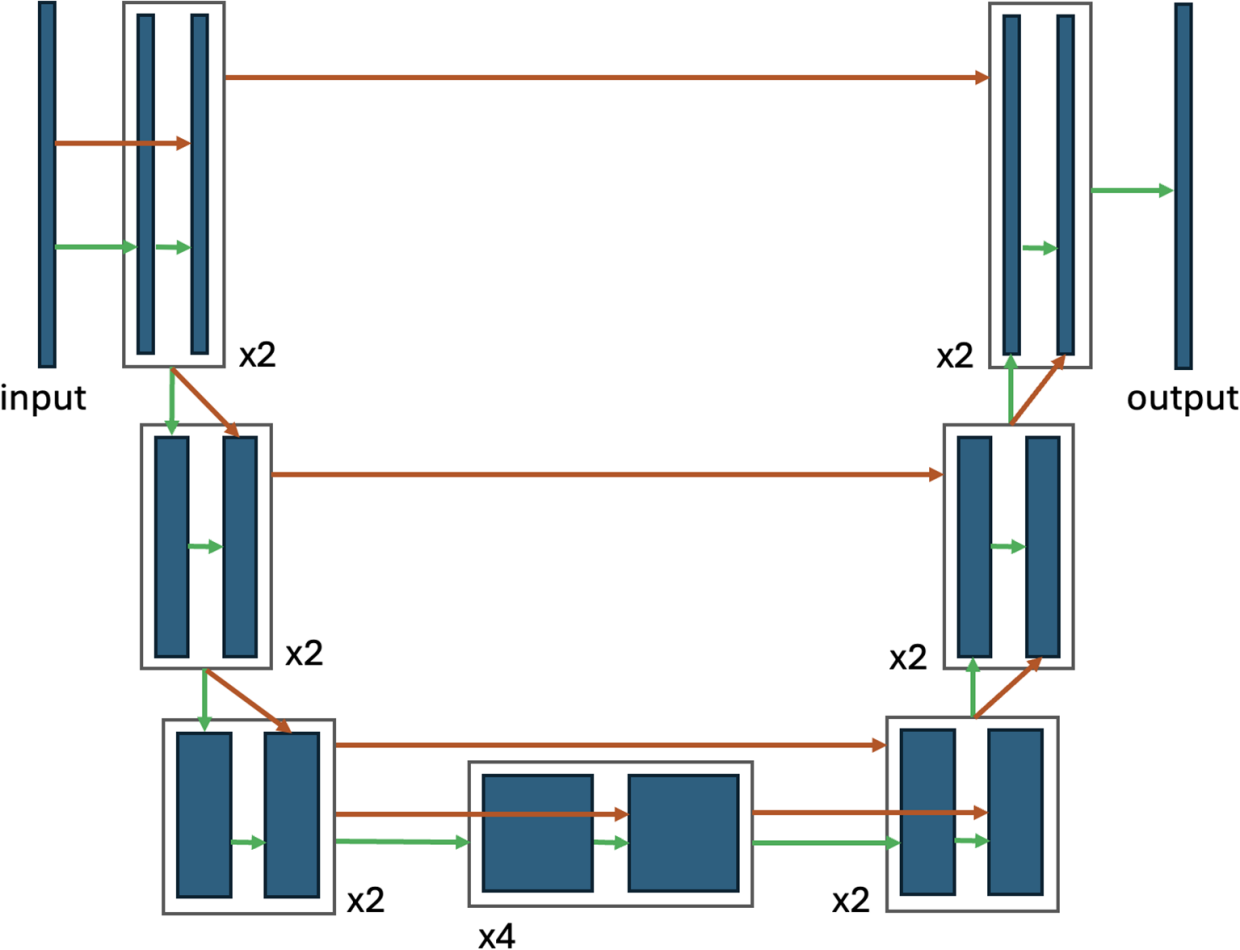
U-Net neural network simplified architecture. This fully convolutional neural network (CNN) is specifically designed for image segmentation applications. The network operates in two distinct pathways, *i.e.*, encoder and decoder. Contracting path (encoder) is a pathway that employs standard convolutional operations to progressively down-sample the input image. This process aims to capture high-level features within the data. Expanding path (decoder) is a pathway that up-samples the extracted high-level features, while simultaneously incorporating precise spatial information preserved from the contracting path. The dark blue rectangles in the diagram represent the feature maps, while the stacked rectangles illustrate the layers of processing applied to these feature maps, the green arrows represent the forward data flow within each block. Crucial spatial information transfer is achieved using skip connections (orange arrows). These connections establish direct links between corresponding layers of identical spatial resolution, ensuring the retention of essential details. Furthermore, the architecture incorporates global skip connections. These connections project feature maps from the lowest resolution of the contracting path to all subsequent processing stages within the expanding path. This ensures that all levels within the network benefit from the complete image context, enhancing segmentation accuracy. By effectively combining high-level feature extraction with precise spatial localization, the U-Net architecture demonstrates its capability to achieve accurate image segmentation tasks even in scenarios with limited training data.

The nuclei segmentation approach in this study is based on the Cellpose method (RRID: SCR_021716) (23). We used the same symmetric, fully convolutional neural network (CNN) architecture called “U-Net” (36), data preprocessing and loss function. The latter is a function that compares the predicted segmentation output with the real one to calculate the prediction error. We adapted the Cellpose method for our data domain and demonstrate here that this adaptation can be done in a “few-shot regime” (using only a small number of labeled data, *i.e.*, expert-annotated images). The Cellpose model was chosen because it was trained on a large and diverse dataset, which gave us a good starting point to adapt the pre-trained neural network to the thyroid epithelial cell data domain. The “U-Net” neural network architecture (**Figure 1**) is a CNN designed for semantic segmentation tasks, particularly in medical imaging. It features a U-shaped structure with a contracting path (encoder) for capturing context and an expansive path (decoder) that preserves spatial information through skip connections, enabling accurate segmentation, even with a limited amount of labeled data. Machine learning (ML) terminology is given in **Supplementary Table 3**.

Twenty-one images of non-treated or nine images of solvent-treated (0.2% DMSO) controls and 34 different inhibitor- or thyroid stimulating hormone (TSH) incubated Nthy-ori 3–1 cell cultures were used as train and test sets, respectively, and in a few-shot fine-tuning ML approach (see **Supplementary Figure 1**), where a small number of images only from the train set of each condition is used. Namely, images of the following experimental treatments were used as datasets for the nuclei segmentation model resulting in the identification of nuclei as representatives of individual cells. Train set: Non-treated controls of two independent experiments, *i.e.*, 13 and 8 images, respectively, and solvent-treated controls of one independent experiment, *i.e.*, 9 images were included. We conducted experiments with two types of train sets: train set A included only data of non-treated controls (21 images), and train set B included only data of solvent treated controls (9 images). The full train set included both A and B sets (see corresponding columns in **Supplementary Figure 1**). Test set: Four independent experiments in which cells were incubated with different cysteine peptidase inhibitors or TSH (see also Ref. 31), *i.e.*, 8, 10, 10, and 6 images, respectively, were included as listed: Nthy-ori_DCG04_E64 (8 images), Nthy-ori_E64 (10 images), Nthy-ori_E64d (10 images), and Nthy-ori_TSH (6 images) (see corresponding columns in **Supplementary Figure 1**). Please refer to **Supplementary Figure 1**, where the images are given with denominations as belonging to the train and/or test set, respectively. It is important to note that this same test set was used to obtain the results of all experiments. For optimizing the segmentation model’s hyperparameters, the Optuna package was used (37). This optimization was performed on train data only *via* k-fold cross-validation, with k = 3. The search for optimal hyperparameters was carried out according to a set of hyperparameters that have the greatest impact in training neural networks for segmentation (21). The list of hyperparameters is as follows: Learning rate, weight decay, batch size, number of epochs, and number of images per epoch. Optimal hyperparameters are given in **Supplementary Table 4**.

In the few-shot setting, we tested the performance of the model when it was trained with support images from each experiment in the train data. In this setting we randomly choose images from each condition from the train sets A and B (first three columns in **Supplementary Figure 1**). The number of chosen images is the parameter of few-shot setting calls to support images number (SIN). For optimizing hyperparameters of the nuclei segmentation model in a few-shot fine-tuning setting, Optuna was also used with k-fold cross-validation and k = 3. In each fold-step, support images were selected randomly three times, and the results were averaged. This allows for increased robustness of the approach. Optimal hyperparameters for the few-shot setting are given in **Supplementary Table 5**.

During the test time, 50 test-launches for each segmentation model experiment type were conducted and the result of an average value for these 50 test-launches with the corresponding standard deviation is reported. Before the training stage, as well as during the testing stage, the input images were pre-processed by resizing them to 512 x 512 pixels, respectively.

Metrics that are used to compare different models on the test set are as follows (see also **Supplementary Table 3** for further ML terminology):

- True Positives (TP) - the model predicted a label (output) that matches correctly as per ground truth.
- False Positives (FP) - the model predicted a label (output), but this was not part of the ground truth (Type I Error).
- False Negatives (FN) - the model does not predict a label (output) but is part of the ground truth (Type II Error).
- Average precision (AP) - TP/(TP + FP + FN).
- Precision - TP/(TP + FP).
- Recall - TP/(TP + FN).
- F1-score - 2 * (Precision * Recall)/(Precision + Recall).
- Image cell number ratio (CNR) - the predicted number of cells in the image divided by the number of cells present in the image as per ground truth. The perfect value of CNR is 1.

### 2.7 Cilia detection approach

The cilia detection approach in this study accurately identifies cilia structures in fluorescence microscopy images using classical image processing techniques. The method unfolds in three main stages: (i) image pre-processing to enhance feature visibility, (ii) potential cilia detection *via* the connected component methodology, and (iii) the refinement process to eliminate false positive objects, ensuring high accuracy of detection.

#### 2.7.1 Image pre-processing

First, we addressed the challenge of cilia detection by selecting the most discernible fluorescence channel (green or red) containing the signal specific to cilia without background noise from other channels in composite TIFF-input images at their original size. To further improve the contrast of cilia structures, we applied Contrast Limited Adaptive Histogram Equalization (CLAHE) (38), known for its effectiveness in improving local contrast in images. This is complemented by Gaussian filtering (39) to smooth the image and reduce background interference. The subsequent top-hat transformation (40), utilizing a disk-shaped structural element, is effective in highlighting small and bright structures with linear features, such as cilia, against a complex background by correcting uneven illumination. Finally, Yen’s thresholding method (41) is applied to contrast potential ciliary structures against the background, based on their intensity, ensuring that the features of interest are distinctly visible for further analysis.

#### 2.7.2 Cilia detection

Following pre-processing, the images are subjected to the connected components algorithm (42) to outline individual cilia candidates. Each detected component is approximated as an ellipse, allowing the derivation of key geometric attributes, namely, length (major axis of an ellipse), thickness (minor axis of an ellipse), area, eccentricity, and perimeter. These attributes serve as quantitative proxies for characteristics of primary cilia, facilitating the identification and quantitative description of their structures. Additionally, these characteristics can be used to filter out false positives, ensuring that only candidates with cilia dimensions above a certain threshold, which distinguishes them from noise, are retained for further analysis.

#### 2.7.3 Refinement process

To reduce false positive detection of primary cilia, which remains a challenge despite thorough preprocessing, we applied additional criteria on the expected length, area, and other quantitative features of cilia. This filtering process ensures that only candidates with dimensions consistent with typical primary cilia are retained for further analysis.

To further refine the determination of cilia lengths, we perform an analysis of cilia skeletons obtained *via* Medial Axis Transform (43). The assumption is that the primary cilium skeletal structure represents the central axis of a filament, thereby providing a more accurate depiction of cilia length and orientation. This method enhances the precision of cilia measurements, ensuring that the detected structures closely match the actual biological features. It is also superior in cilia detection if those are particularly long, bent, or curved.

This approach thus integrates pre-processing techniques, rigorous extraction algorithms, and refined measurement processes to achieve high accuracy in detecting and analyzing primary cilia in microscopy images.

#### 2.7.4 CU Cilia output features (definitions)

CU Cilia reports per-cilia descriptors beyond commonly used frequency and length measures, facilitating benchmarking and downstream analysis.

Notably, cilia length determination can be achieved by either of the measures for major axis length, perimeter, and/or skeleton length.

Per-cilia features:

- Area [pixels²]: The area of each primary cilium.
- Perimeter [pixels]: The total length of the circumference of a primary cilium.
- Eccentricity [0-1]: The degree to which a cilium deviates from a perfect circle, representing its elongation. 0 indicates a perfect circle, and 1 represents a degenerate case in which the ellipse becomes a line segment.
- Form Factor: A quantitative measure of the primary cilium’s shape, indicating how closely it approximates a circle, calculated using the formula:

$$\text{Form Factor }=\;4\;\pi \times \text{ Area}/\text{Perimete}{\text{r}}^{2}$$

- Axis Minor Length [pixels]: The length of the fitted ellipse major axis.
- Axis Major Length [pixels]: The length of the fitted ellipse minor axis.
- Skeleton Length [pixels]: The length of the cilium centerline.

The list of these characteristics can be customized if needed (see CILIA_FEATURES parameters in **Table 1**).

**Table 1:** CU Cilia configurable parameters.

Parameter name	Description	Default value
CILIA_GAUSS_SIGMA	Standard deviation for the Gaussian kernel: specifies the level of image smoothing applied, with larger sizes resulting in greater smoothing.	8
CILIA_TOPHAT_RADIUS	Top Hat Transformation Radius: Defines the radius used to enhance bright features in the image, making them more prominent.	13
CILIA_MIN_LENGTH	Minimum Cilia Length [pixels]: Sets the threshold for detecting the shortest cilia, with lengths below this value being ignored.	14
CILIA_MIN_AREA	Minimum Cilia Area [pixels^2^]: Sets the threshold for detecting the smallest cilia by area, with areas below this value being ignored.	256
CILIA_MIN_ECCENTRICITY	Minimum Cilia Eccentricity: Sets the threshold for detecting cilia based on their eccentricity. Only cilia with an eccentricity value greater than or equal to this threshold are retained, effectively excluding structures that are too close to a perfect circle and ensuring the selection of elongated cilia.	0.46
CILIA_MIN_PERIMETER	Minimum Cilia Perimeter [pixels]: Sets the threshold for detecting the smallest cilia by perimeter, with perimeters below this value being ignored.	67
EXCLUDE_EDGE_CILIA	Flag indicating whether to exclude cilia located at the edges of the analyzed area. When set to True, edge cilia are omitted from processing.	False
CILIA_COLOR	Cilia Color Channel: Specifies which color channel(s) to use for identifying cilia in the RGB images, with options “red,” “green,” or “red+green”.	“red+green”
CILIA_FEATURES	List of cilia features to calculate and visualize.	‘[“area”, “perimeter”, “eccentricity”, “form_factor”, “axis_minor_length”, “axis_major_length”, “skeleton_length”]’
CELLPOSE_FLOW_THRESHOLD	Flow Threshold: Sets the error threshold for the nuclei segmentation model, controlling the accuracy of flow detection.	0.4
CELLPOSE_MIN_SIZE	Minimal Nuclei Size [pixels^2^]: Sets the threshold for discarding small regions of interest (ROIs) during nuclei segmentation, ensuring only sufficiently large nuclei are considered, while apoptotic bodies or mitotic spindles are below this threshold.	15
EXCLUDE_EDGE_NUCLEI	Flag indicating whether to exclude nuclei positioned at the boundaries of the analysis region. When set to True, edge nuclei are omitted from the analysis.	False

### 2.8 Aggregated features derived from nuclei and cilia

Moreover, the combination of results from nuclei segmentation and cilia detection in the CU Cilia approach enables determination of complex features critical for the analysis of high-content images taken from biological samples. The full list of aggregated characteristics includes:

- Nuclei numbers: The total number of nuclei present in an image representing the number of non-dividing cells. Note, cells in mitotic division and apoptotic bodies are not included.
- Area of Nuclei: The ratio of the area covered by nuclei to the total area of the image; thus, it is indicative of the cell culture confluence.
- Individual Nucleus Area Ratio: Ratio of the area of individual nuclei to the total area of nuclei in the respective image. This measure gives information on the nuclear size (area) distribution in the population of all nuclei in a respective image.
- Cilia Numbers: The total number of primary cilia in the image.
- Percentage of Cilia within Cells: The proportion of cilia located within cells relative to the total number of cilia. This measure indicates whether cilia are distant from nuclei due to, *e.g.*, cell polarization or cell damage.
- Mean Nearest Center Mass Distance: The average distance from the center of each cilium to the center of the nearest nucleus.
- Mean Nearest Boundary Distance: The average distance from the boundary of each cilium to the boundary of the nearest nucleus, indicating which primary cilium belongs to which cell.

These characteristics can provide deep insight into cellular architecture of physiological and pathological states of cells (see Section 3).

### 2.9 CU Cilia image analysis pipeline

**Figure 2:**
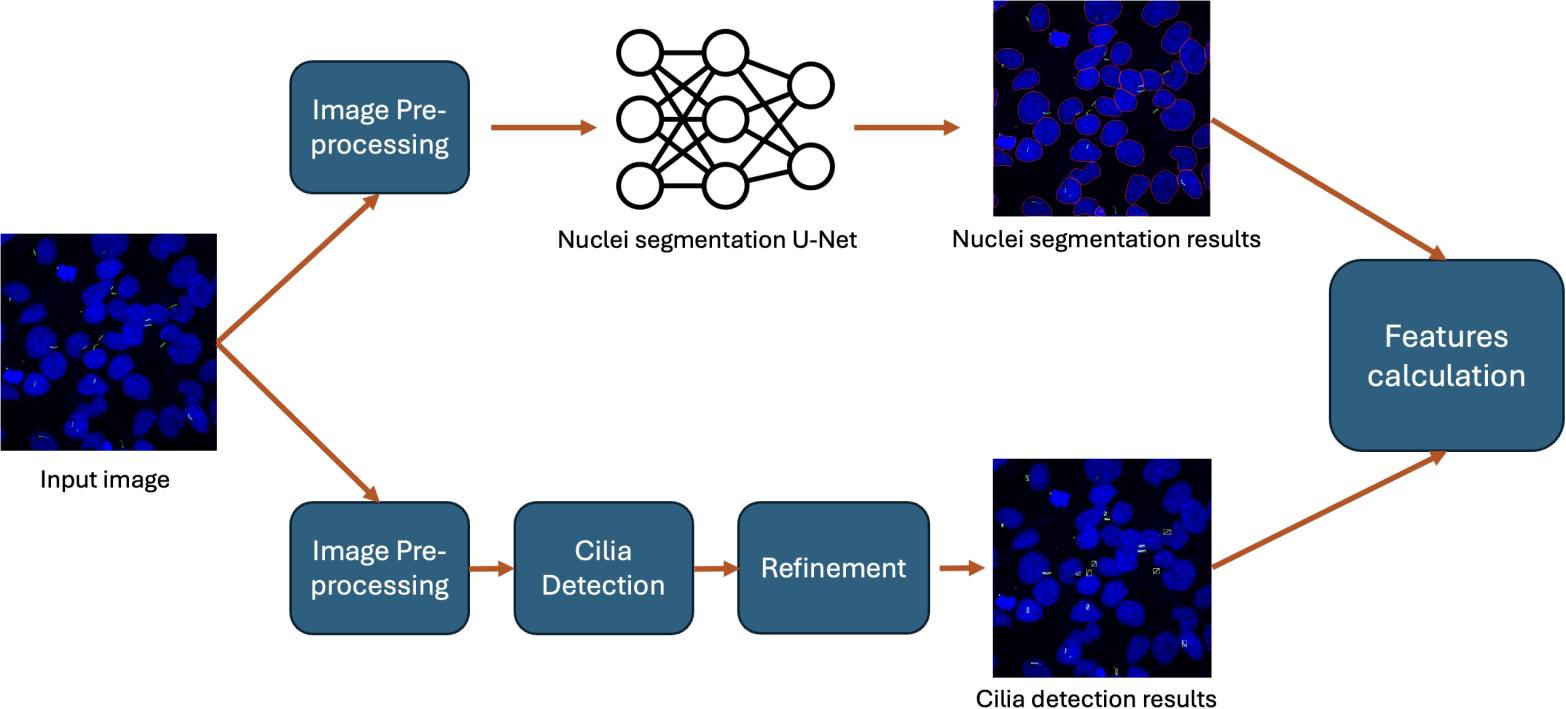
CU Cilia image analysis pipeline. The CU Cilia image processing pipeline consists of two branches, namely, nuclei segmentation and cilia detection. In the nuclei segmentation branch the input image is resized and fed to the input of the segmentation U-Net (fine-tuned from the Cellpose model). In the cilia detection branch the input image sequentially goes through the stages of pre-processing, cilia detection and refinement. The results of these two branches are aggregated in a features extraction module to produce the final output.

To analyze input images, CU Cilia launches nuclei segmentation and cilia detection pipelines described above (see Sections 2.6 and 2.7). The obtained results are aggregated in the feature calculation module, which is then used to quantitatively determine the characteristics of the cells in the input image for further data analysis. These characteristics provide deep insight into cellular architecture as they describe the state of the cells comprehensively. Calculation of these features completes the CU Cilia image analysis pipeline (**Figure 2**).

CU Cilia is implemented on the Constructor Platform, which prioritizes user-friendliness while allowing customization. Users can adjust various parameters to optimize cilia detection for their specific datasets. The names, descriptions, and default values for these parameters are listed in **Table 1**.

The code of CU Cilia and related experiments can be found in the Constructor Platform (https://constructor.app/platform/research/public/project/cu_cilia), or in the following repository on GitHub: https://github.com/andrewbo29/cu_cilia.

### 2.10 Statistical analysis

Nuclei counts and the data quantifying primary cilia features are shown as means ± standard deviations from pilot or independently repeated experiments. Individual data points are represented by circles or dots in addition to the bar charts, allowing for identification of individual values.

GraphPad Prism™ (version 8.0.2; RRID: SCR_002798, GraphPad Software Inc., San Diego, CA, USA) was used throughout. Means and standard deviations were determined by performing descriptive statistics of datasets.

Gaussian distribution was analyzed by Anderson-Darling, D’Agostino & Pearson, Shapiro-Wilk and Kolmogorov-Smirnov tests. When these normality tests were not passed by any of the datasets that were to be compared, we assumed a non-equal distribution and performed non-parametric Kruskal-Wallis tests as an alternative to a one-way ANOVA, and additionally Dunn’s multiple comparison *post hoc* test. P-values below 0.05 were considered statistically significant and are indicated as * for p<0.05, ** for p<0.01, *** for p<0.001, and **** for p<0.0001.

## 3 Results

### 3.1 Necessity to set up an algorithm for detection of nuclei and primary cilia of Nthy-ori 3–1 cells *in vitro*

**Figure 3:**
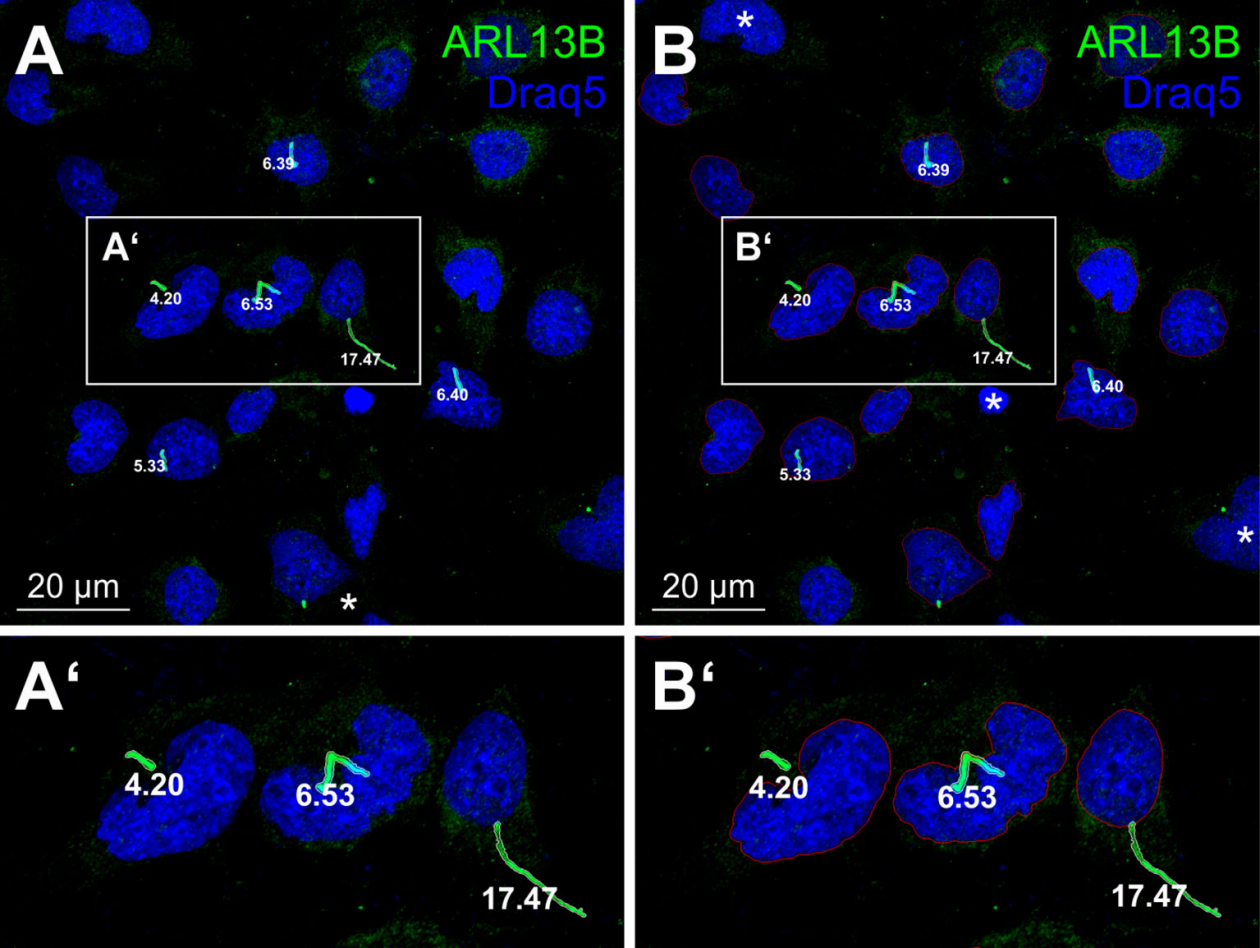
Primary cilia of human thyroid epithelial cells *in vitro*. Confocal fluorescence micrographs of cultured human thyroid epithelial cells upon fixation with paraformaldehyde and methanol, and immunolabelling with antibodies against the cilia marker ARL13B (green) and counter-staining of nuclear DNA with Draq5™ (blue). Boxed areas in **(A, B)** are shown in higher magnification in A’ and B’, respectively. Major axis lengths of primary cilia **(A, A’)** are annotated in µm next to the respective cilia, demonstrating a wide length range of well-extended or bent cilia. Detected nuclei are surrounded by a red line **(B, B’)**. Asterisks denote apoptotic bodies and nuclei touching the image borders, which are excluded from CU Cilia detection and enumeration.

From our previous study (31), we reasoned that the outcomes of, *e.g.*, cysteine peptidase inhibitor treatments must appreciate the number of intact cells. This is well-approachable by counting nuclei with a typical oval to kidney-like shape, but can be particularly challenging in dense, confluent cell cultures. An even more precise measure of cell integrity could be provided by determining the number of primary cilia and their lengths in a population of non- and drug-treated cells. Here, challenges arise because primary cilia are not always straight structures but may be bent or curved (**Figure 3**).

Thus, for segmentation of the structures of interest, namely, nuclei and primary cilia, we have set up CellProfiler™ pipelines (see **Supplementary Tables 1**, **2**) that allow determining the respective parameters upon fine-tuning of the settings. The results are promising, because the outlines of nuclei and primary cilia follow the Draq5™- and anti-ARL13B-stained structures, respectively, with good precision in non-confluent culture conditions, while confluent cell cultures pose a challenge in identifying individual nuclei without manual editing. Thus, adjusting the parameters in individual modules of the pipelines requires expert knowledge and substantial time depending on the prior training of the respective users.

**Figure 4:**
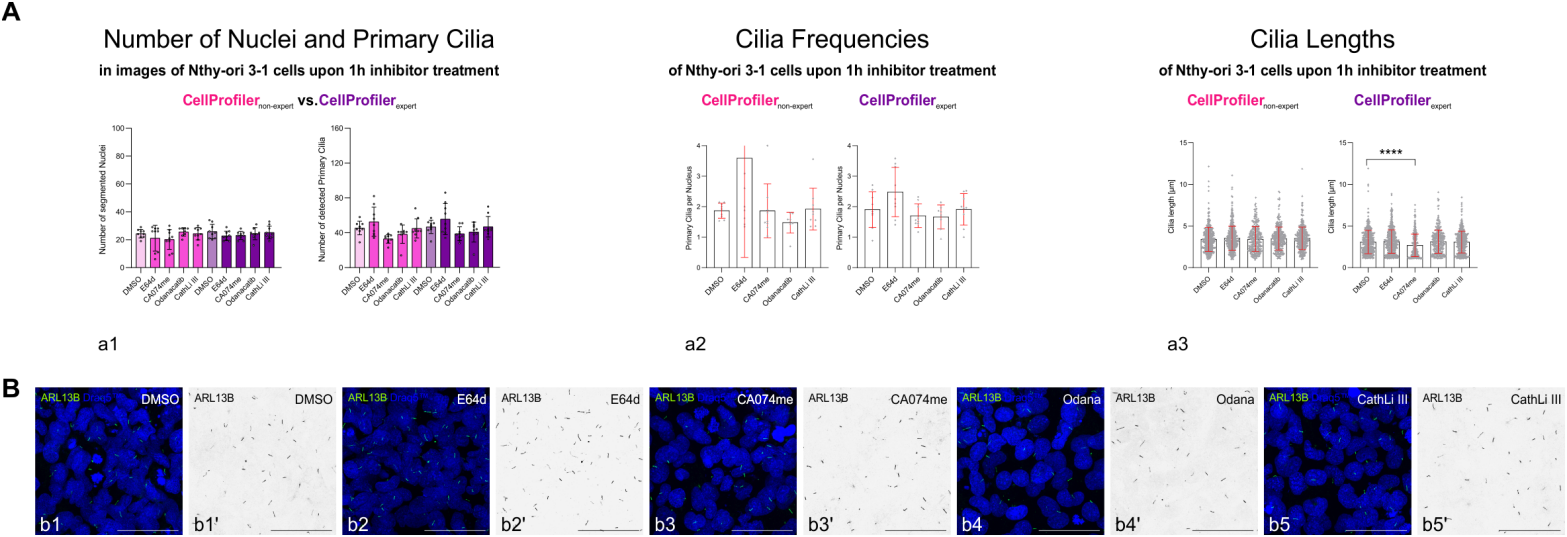
Detection of nuclei and primary cilia of human thyroid epithelial cells *in vitro* using CellProfiler™ pipelines. **(A)** Bar charts comparing the numbers of detected nuclei and primary cilia **(a1)**, cilia frequencies **(a2)** and cilia lengths **(a3)** as revealed by CellProfiler™ pipelines set up by non-expert (pink, left panels) and expert users (violet, right panels). Mean values ± standard deviations are displayed in the bar charts with individual data points indicated by circles or dots **(a1–a3)**. Statistical analysis was by Kruskal-Wallis multiple comparisons tests; levels of significance are indicated as **** for p<0.0001. Note that the ranges of measurements differed between non-expert and expert determinations, while the 1 h-treatments with broad-spectrum (E64d) or specific cysteine peptidase inhibitors (CA074me, Odanacatib, CathLi III) did not usually differ from controls (DMSO). **(B)** Merged channel confocal laser scanning micrographs depicting ARL13B-positive primary cilia (**B**, green in **b1–b5**; black in **b1’–b5’**) and Draq5™-stained nuclei (**B**, blue in **b1–b5**) of DMSO-treated controls **(b1, b1’)** or Nthy-ori 3–1 cell cultures treated with broad-spectrum **(b2, b2’)** or specific inhibitors of cathepsin B **(b3, b3’)**, cathepsin K **(b4, b4’)** or cathepsin L **(b5, b5’)**, respectively. Note that corresponding single channels of anti-ARL13B-positive primary cilia are shown in inverted contrast **(b1’–b5’)** for clarity. Scale bars represent 50 µm. Identical images were analyzed with the two different pipelines with n=9 technical replicates, except n=8 for Odanacatib-treated cells.

For determination of differences between 1 h cysteine peptidase inhibitor-treated Nthy-ori 3–1 cell cultures, data analyses with pipelines set up by users of different expertise levels (**Figure 4**; **Supplementary Figures 2**, **3**) yielded comparable results. However, pipeline set up by differently experienced users resulted in data spanning different ranges (**Figure 4A**, cf. standard deviations of left and right panels, respectively), which only occasionally pointed to differences between cysteine peptidase inhibitor-treated *versus* control cells (**Figures 4A, a1, a2, a3, 4B**).

Due to the small differences detected upon cysteine peptidase inhibitor treatment and results varying among different users, we sought to prolong inhibitor treatment (see below) and, first, to involve machine learning (ML) to put in place algorithms by establishing CU Cilia that would reproducibly and reliably segment nuclei and detect primary cilia of thyroid epithelial cell cultures, independent of the users’ expertise.

### 3.2 Segmentation for automated detection of nuclei by CU Cilia outperforms Cellpose

The nuclei segmentation experiments were carried out using the dataset composition detailed in Section 2.6 and **Supplementary Figure 1**. In summary, training utilized control images from non-treated and solvent-treated Nthy-ori 3–1 cell cultures, whereas the test set comprised images from four independent inhibitor- or TSH-treatment experiments. The main idea of such train and test dataset splitting is to validate the hypothesis that it is sufficient to train the segmentation model only on non-treated or solvent-exposed control Nthy-ori 3–1 cell cultures before extending the results of the nuclei segmentation model on images taken from cysteine peptidase inhibitor or otherwise treated cells, which might get affected by any treatment regarding their cellular or nuclear shapes. The segmentation model trained only on non-treated controls (train set A) is denoted as Model A, while the segmentation model trained on both non-treated control and solvent-exposed control (train set A and train set B) is denoted as Model A+ B. The segmentation dataset is publicly available to allow reproduction (see file “nuclei_dataset.zip” in https://zenodo.org/records/14871212). This dataset includes original images from train and test sets, as well as manually labeled segmentation masks (in npy- format) that were used to train and test our nuclei segmentation model. Importantly, we also wanted to explore whether training the nuclei segmentation model is possible with only a few images, *i.e.*, in the few-shot regime applied in this study. In general, the CU Cilia approach (this study) is compared with results achieved by CellProfiler™ (22) and Cellpose (21, 23).

The CU Cilia approach outperformed the nuclei segmentation results achieved using Cellpose (**Table 2**, row “Cellpose”) regarding all metrics and segmentation model experiment types (**Table 2**, rows “Model A”, “Model A + B”). The best results were achieved when the model was trained on the train set type A (untreated cells). This model outperformed the original Cellpose model by 4.8% for AP at 0.5 IoU, 5.5% for AP at 0.75 IoU, and 0.014 for Mean |1 – CNR|. The percentages are calculated as the differences between the values in rows “Model A” subtracted by values in rows “Cellpose” (**Table 2**).

**Table 2:** Comparison of experiments based on average metrics obtained over the entire test set.

Segmentation model experiment type	AP at 0.5 IoU (high is better)	AP at 0.75 IoU (high is better)	Mean |1 - CNR| (lower is better)
Cellpose	0.794	0.651	0.031
Model A	**0.842 ± 0.002**	**0.706 ± 0.003**	**0.017 ± 0.003**
Model A + B	0.838 ± 0.001	0.705 ± 0.001	0.039 ± 0.001
Model A (SIN = 5)	0.837 ± 0.001	0.703 ± 0.002	0.034 ± 0.004
Model A + B (SIN = 5)	0.837 ± 0.002	0.703 ± 0.003	0.034 ± 0.005

The table displays the average values with standard deviations after 50 test launches of the corresponding experiment.

Bold values means the best metrics.

The CU Cilia method in the few-shot fine-tuning setting achieved comparable results with support images number (SIN) equal to 5 on non-treated (A) and on both non-treated and treated (A + B) train datasets (**Table 2**, rows “Model A (SIN = 5)”, “Model A + B (SIN = 5)”). However, the results were worse than for the model trained on the full train set A but still outperformed the original Cellpose by 4.3% for AP at 0.5 IoU, and 5.2% for AP at 0.75 IoU (percentages are calculated as the differences between the values in rows “Model A (SIN = 5)” subtracted by values in rows “Cellpose” in **Table 2**). It should be emphasized that the same test set was used for both the few-shot fine-tuning and other experimental settings. The only source of randomness came from selecting support images from the respective training sets for each run. As **Table 2** reports the average performance over 50 runs, the results can be regarded as statistically robust.

The separated results for each cell culture condition from the test set are given in **Supplementary Tables 6**-**9**.

**Figure 5:**
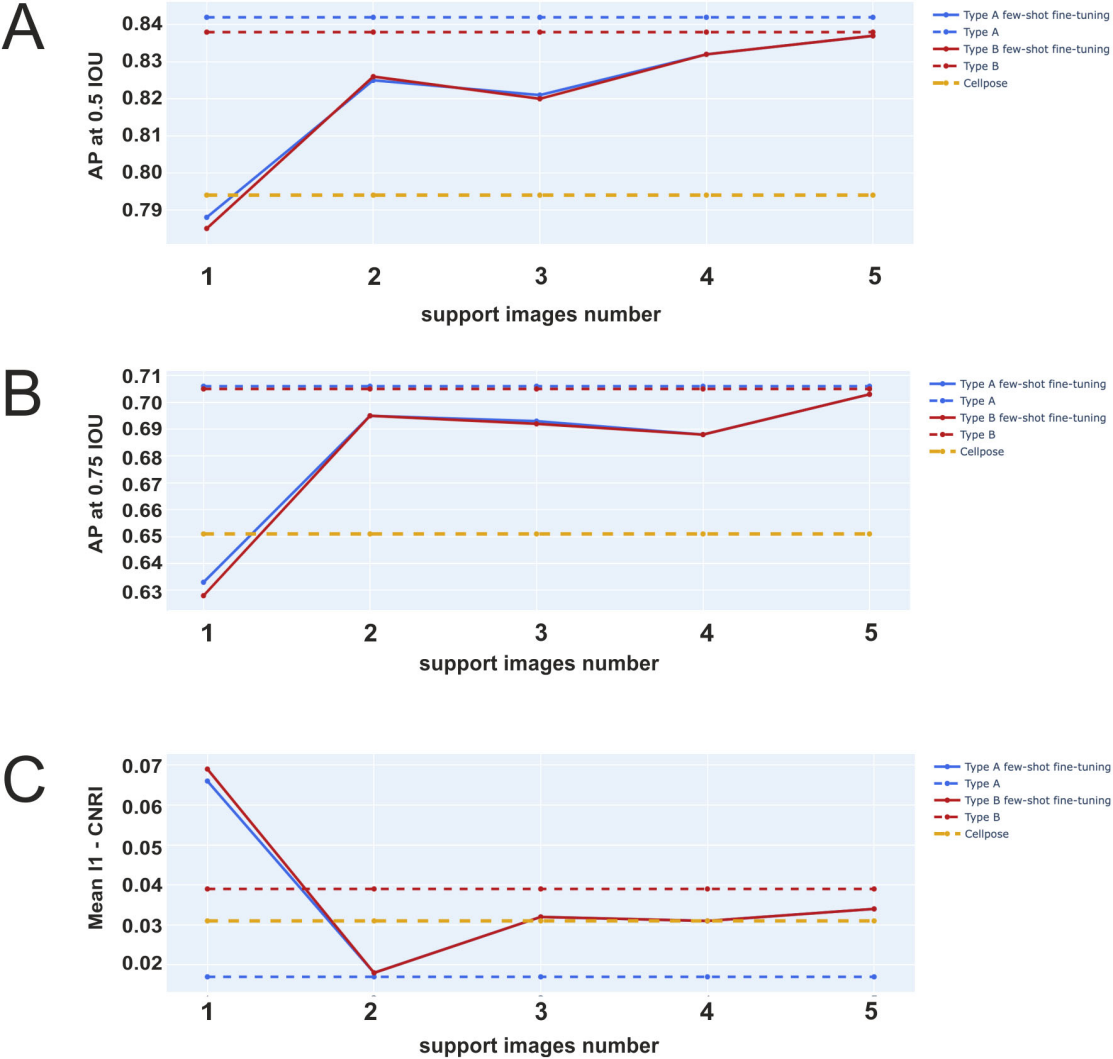
AP few-shot fine-tuning experiment. AP at 0.5 IoU (high is better) for the few-shot fine-tuning experiment **(A)**. AP at 0.75 IoU (high is better) for the few-shot fine-tuning experiment **(B)**. Absolute value of Mean CNR (lower is better) for the few-shot fine-tuning experiment **(C)**. Each data point is the average over 50 launch results on the test set for the model trained on support images from each experiment in the train set.

Increasing SIN increases AP for both Model A and Model A + B (**Figures 5A, B**). For SIN = 5, the mean CNR is slightly lower than that of the original Cellpose model, but it achieves better results than for SIN = 2 (**Figure 5C**).

**Figure 6:**
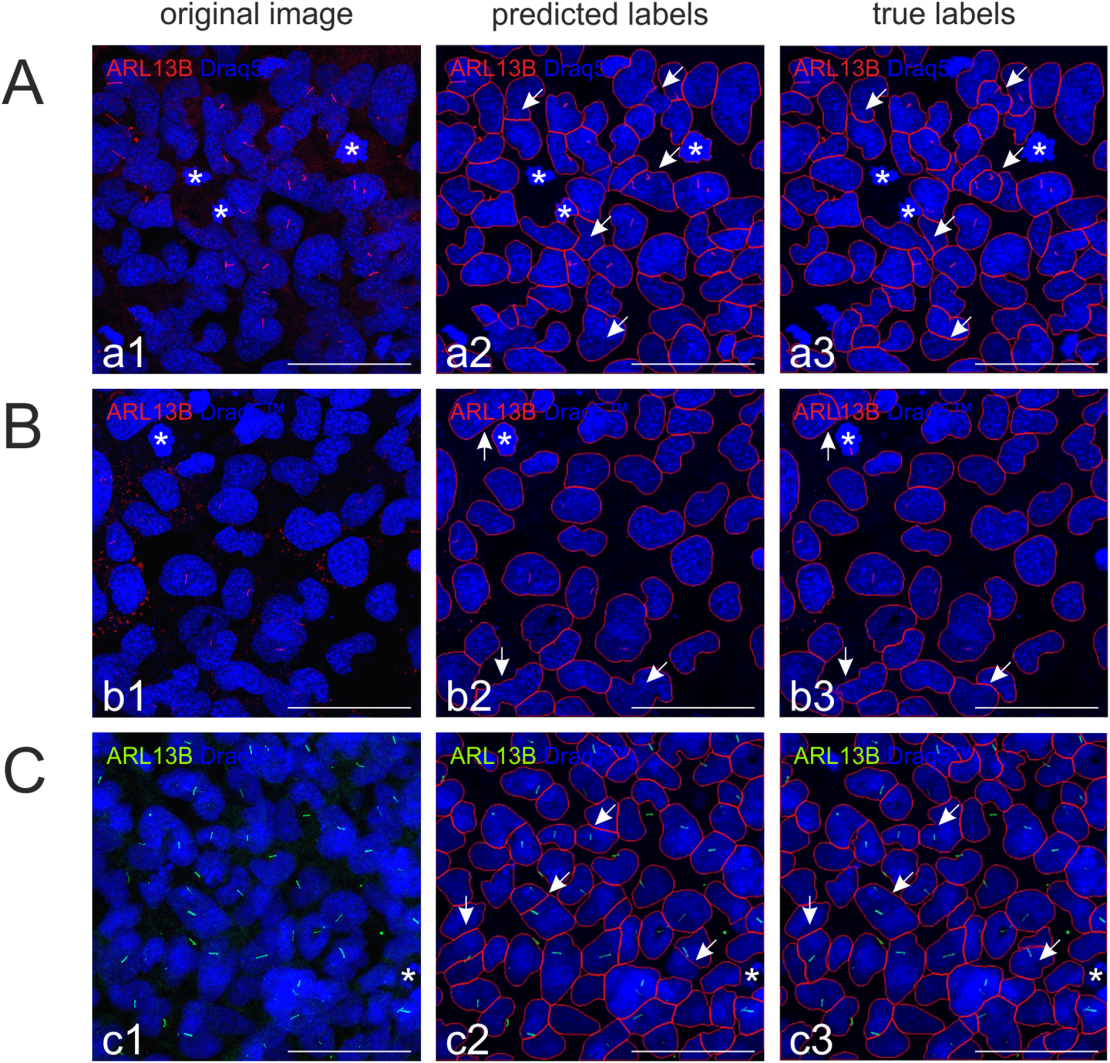
CU Cilia’s model segmentation masks on images from the test sets. **(A) **CU Cilia’s model A segmentation masks on the image from the test set (Nthy-ori_E64d_image 5); AP at 0.5 IoU = 0.898, AP at 0.75 IoU = 0.755; predicted cell number: 46, and ground truth cell number: 47. **(B) **CU Cilia’s model segmentation masks on the image from the test set (Nthy-ori_E64d_image 10); AP at 0.5 IoU = 0.797, AP at 0.75 IoU = 0.614; predicted cell number: 72, and ground truth cell number: 70. **(C) **CU Cilia’s model segmentation masks on the image from the test set (Nthy-ori_TSH_image 6); AP at 0.5 IoU = 0.763, AP at 0.75 IoU = 0.726; predicted cell number: 83, and ground truth cell number: 81. Input images (left; a1, b1, c1), CU Cilia model prediction (center; a2, b2, c2), and ground truth segmentations are displayed as indicated (right; a3, b3, c3). Scale bars represent 50 µm, asterisks denote mitotic cells, and arrows point to differences between predicted-labels and true-labels.

Results of the few-shot fine-tuning setting demonstrated that it is possible to label a few images (5 images for each experiment) in CU Cilia to achieve good nuclei segmentation results that outperform the original Cellpose model. Visualization of the segmentation results demonstrates that nuclei segmented by CU Cilia were well-defined and comparable to manually circumscribed nuclei (by an expert), while CellProfiler™ pipelines missed detecting some of the nuclei (**Figure 6**).

The best results are achieved when non-treated control cells are chosen to train the model (see **Table 2**; **Supplementary Tables 6**-**9**), highlighted numbers in boldface). It is further important to note that CU Cilia is not set out to detect cells in mitosis (**Figure 6**, asterisks), *i.e.*, when the nuclear envelope is broken down and chromosome condensation took place, although these stages are stained with Draq5™, too. However, such mitotic figures do not have an oval shape that is characteristic for nuclei in telophase and cytokinesis, or for interphase nuclei. While mitotic figures can be detected by CellProfiler™, they are typically filtered out by range-determination of object sizes (see Section 2.5).

### 3.3 Cilia detection by CU Cilia in comparison with CellProfiler™

The overall objective of this study was to conduct a comparative analysis between the frequencies as well as lengths and further structural parameters of primary cilia that extend from non-treated and cysteine peptidase inhibitor-treated Nthy-ori 3–1 cells, such as generated by CellProfiler™ (22, 31) and by our newly developed method, CU Cilia. We selected a set of 16 images, which are publicly available (see file “cilia_dataset” in https://zenodo.org/records/14871212), divided into three categories (see also **Supplementary Figure 4**) as explained below.

- Expert-annotated data, *i.e.*, manually labeled, which we used for optimizing cilia detection hyperparameters. This subset included 6 images from various experiments and conditions.
- “Easy” cases consisting of 5 images annotated using CellProfiler™, which the expert deemed to be perfectly labeled.
- “Difficult” cases consisting of 5 images also annotated using CellProfiler™, where the expert identified labeling errors, such as non-detected or incorrectly detected cilia.

Our evaluation aimed at benchmarking the performance of CU Cilia *versus* CellProfiler™ in primary cilia description by using the results of the latter approach as a reference. After optimizing parameters on the expert-annotated dataset, with the optimal settings summarized in **Table 1** (in the “Default Value” column), we applied the CU Cilia algorithm to the “easy” and “difficult” cases of the dataset. As demonstrated in **Table 3**, CU Cilia achieved high precision, recall, and F1-scores for all “easy” test images, indicating that its results were closely aligned with those produced by CellProfiler™. For the “difficult” cases, CU Cilia exhibited robust performance, further confirming its accuracy. For the “easy” cases, CU Cilia provides a range of data (**Table 4**), which can be further extended to even more thoroughly describe architectural features of primary cilia (see below).

**Table 3:** Test images (Nthy-oriZ_untreated) cilia detection metrics at IoU 0.5.

Test image	Case type	Precision (higher is better)	Recall (higher is better)	F1-score (higher is better)
Nthy-oriZ_untreated image 2	Easy	0.875	1.000	0.933
Nthy-oriZ_untreated image 3	Easy	0.909	1.000	0.952
Nthy-oriZ_untreated image 4	Easy	0.875	0.778	0.824
Nthy-oriZ_untreated image 6	Easy	1.000	1.000	1.000
Nthy-oriZ_untreated image 10	Easy	1.000	1.000	1.000
Nthy-oriZ_untreated image 1	Difficult	0.571	0.889	0.696
Nthy-oriZ_untreated image 5	Difficult	0.789	0.882	0.833
Nthy-oriZ_untreated image 7	Difficult	0.786	0.917	0.846
Nthy-oriZ_untreated image 8	Difficult	1.000	1.000	1.000
Nthy-oriZ_untreated image 9	Difficult	0.882	1.000	0.938

**Table 4:** Properties of nuclei and cilia for “easy” cases, *i.e.*, dataset “Nthy-oriZ_untreated”.

Test image	Nuclei number	Area of nuclei [%]	Individual nucleus area ratio [%]	Cilia number	Cilia within cells [%]	Mean nearest center-of-mass distance [px]	Mean nearest boundary distance [px]
Image 2	46	42.77	0.93	8	87.5	146.48	28.34
Image 3	39	34.29	0.88	11	54.55	179.95	41.83
Image 4	50	27.7	0.55	8	50.0	126.0	40.98
Image 6	67	41.95	0.63	21	90.48	112.63	39.9
Image 10	37	23.38	0.63	9	55.56	126.21	24.32

**Figure 7:**
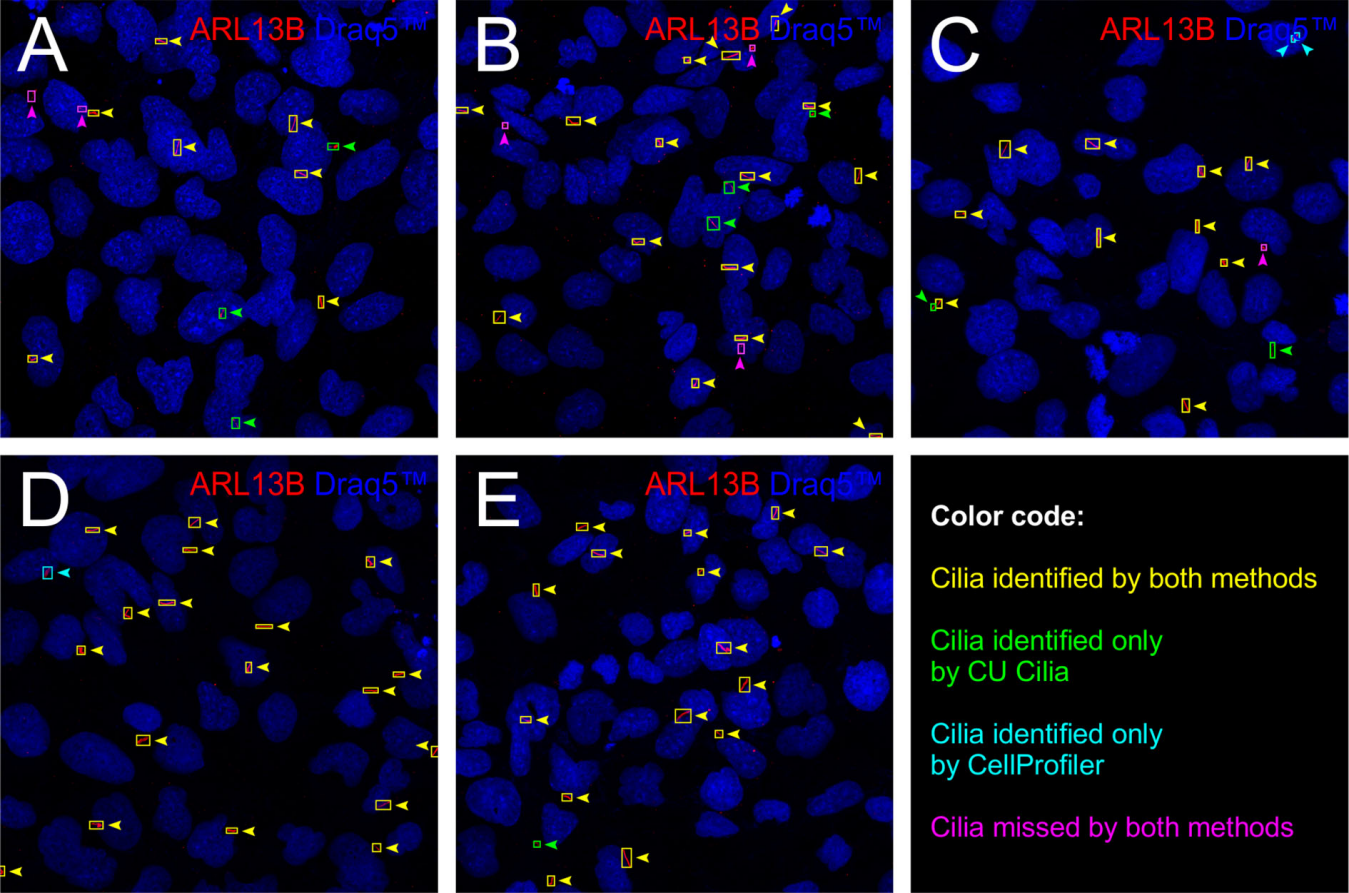
Comparative primary cilia detection results on “difficult” cases. Image 1 **(A)**, Image 5 **(B)**, Image 7 **(C)**, Image 8 **(D)**, and Image 9 **(E)** of the difficult cases dataset were annotated with boxes to indicate detected cilia. Green boxes indicate cilia identified exclusively by CU Cilia, blue boxes denote cilia identified solely by CellProfiler™, yellow boxes denote cilia identified by both methods, and purple boxes indicate cilia missed by both methods. This color-coded visualization scheme underscores effectiveness and limitations of each detection method.

To more comprehensively illustrate similarities and differences in cilia detection by CellProfiler™ and CU Cilia, we further analyzed example images from the “difficult” cases. Visual comparison shows the detection capabilities of both methods using specific color codes (**Figure 7**). An overlay with yellow boxes indicates primary cilia detected by both methods, whereas green boxes highlight primary cilia detected exclusively or more accurately by CU Cilia, and blue boxes denote primary cilia identified exclusively or with greater precision by CellProfiler™. The abundance of yellow boxes, *i.e.*, enclosing cilia recognized equally well by both methods, confirms the reliability of the CU Cilia algorithm in matching the overall precision of CellProfiler™. Particularly noteworthy are the purple boxes, which mark primary cilia missed by both methods, indicating potential areas for improvement in both algorithms. Overall, CU Cilia performs comparably to CellProfiler™ in detecting cilia, while significantly reducing the time required for software set up and fine-tuning.

**Figure 8:**
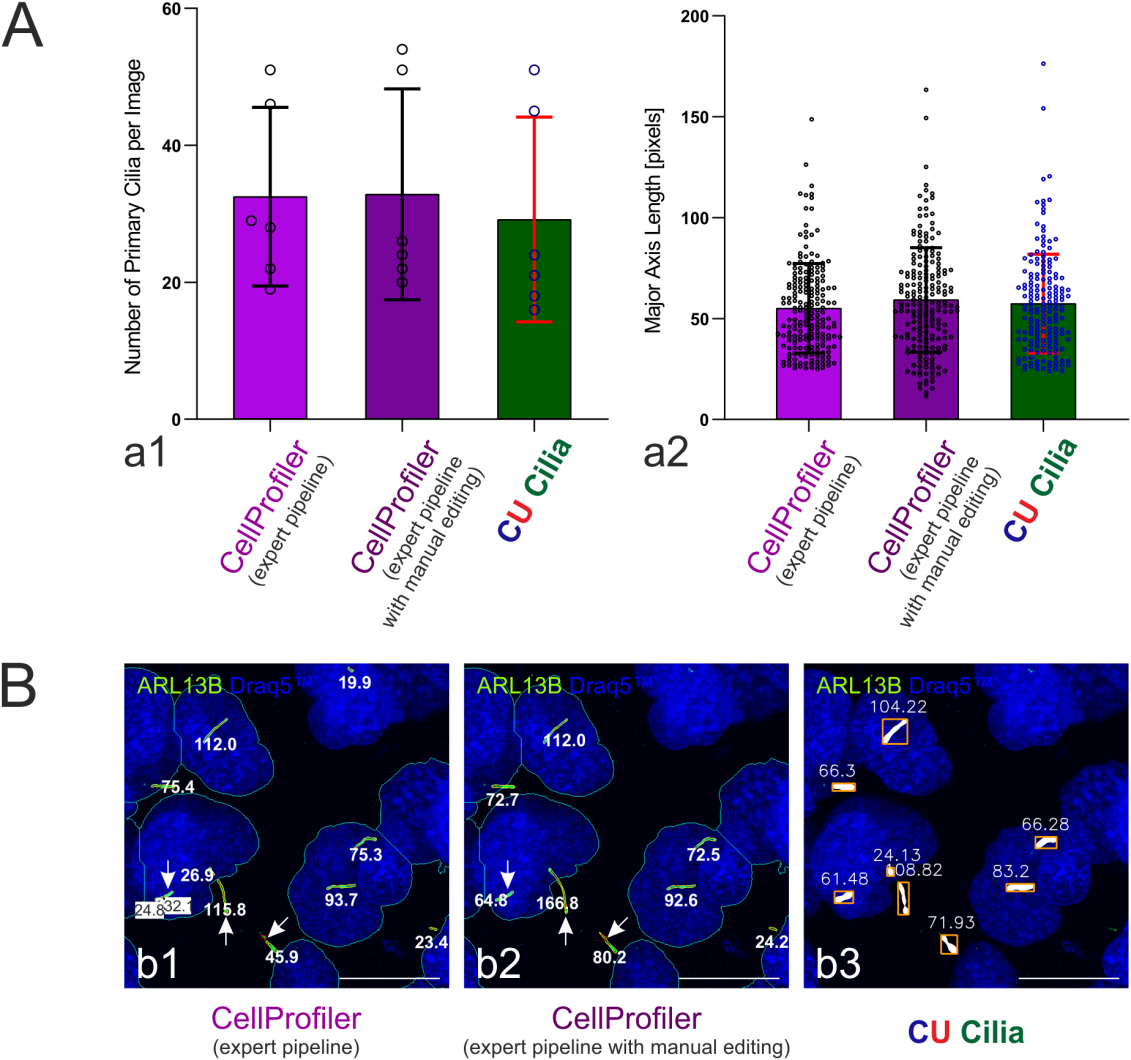
Comparison of cilia numbers and major axis lengths. **(A)** Determination of cilia numbers per image and major axis length in pixels as achieved by CellProfiler™ (expert user pipeline) without (light violet, left) or with manual editing (violet, middle) *versus* CU Cilia (green right) using the parameter settings, *i.e.*, Cilia_Tophat_Radius = 13, Cilia_Gauss_Sigma = 8, Cilia_Min_Length = 10, Cilia_Min_Area = 25, Cilia_Min_Eccentricity = 0.46, Cilia_Min_Perimeter = 67 and Exclude_Edge_Cilia = True. Identical image sets (n = 6) of random choice were analyzed; the numbers of identified and measured primary cilia per image and per analysis method were 32, 34 and 35, summing up to n = 195, n = 197 and n = 175 in all 6 images, with an average length of 55.1 ± 22.2, 59.3 ± 25.8 and 57.4 ± 24.5 pixels, respectively. No statistical significance was detected. **(B)** Note that primary cilia with interrupted appearance need special attention (arrows) as obvious from confocal laser scanning micrographs in which detected cilia are outlined with length data annotations at the side. Scale bars represent 20 µm.

It is important to keep in mind that interrupted cilia structures are particularly challenging to identify as one object and typically require manual editing (**Figure 8**).

Extended per-cilia metrics and aggregated readouts are defined in Methods (see Sections 2.7.4 and 2.8) and detailed below.

### 3.4 Primary cilia and cysteine cathepsins of thyroid epithelial cells

CU Cilia functionalities and capabilities were tested next in context with cysteine cathepsin activities based on our previous investigations on thyroid epithelial cells (30, 31).

**Figure 9:**
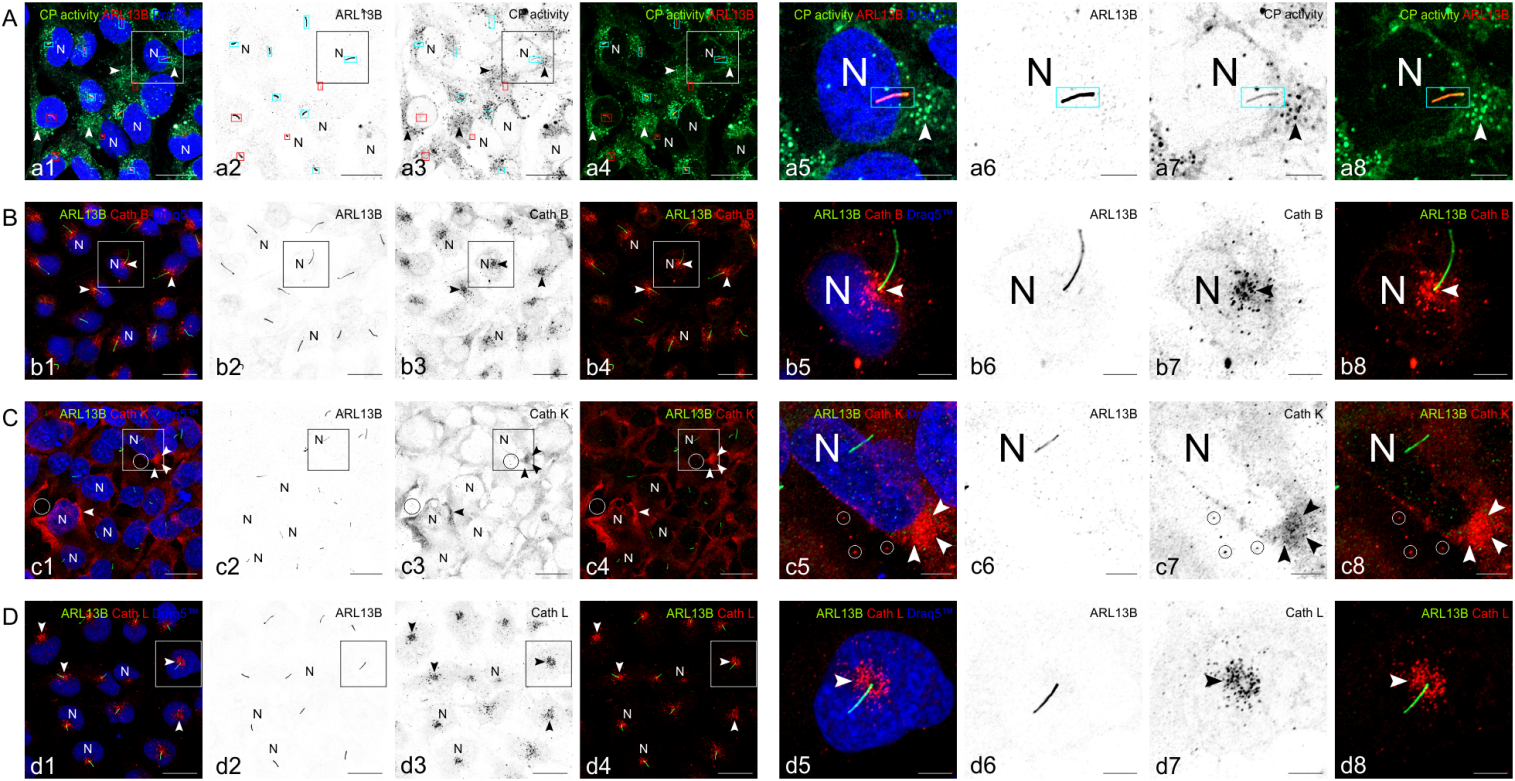
Cysteine peptidase activity at primary cilia of human thyroid epithelial cells *in vitro*. Confocal fluorescence micrographs of cultured human thyroid epithelial cells upon incubation with the cysteine peptidase-specific activity-based probe DCG-04 for 1 h (**A**, green), fixation with paraformaldehyde and methanol, and immunostaining with antibodies against the cilia marker ARL13B (**A**, red; **B-D**, green). Non-treated controls were immuno-stained with antibodies specific for ARL13B (**B-D**, green) and cathepsin B (**B**, red), cathepsin K (**C**, red) or cathepsin L (**D**, red). Counter-staining of nuclear DNA was with Draq5™ (a1, a5, b1, b5, c1, c5, d1, d5, blue; N, nuclei). For better contrast, the single fluorescence channels are displayed as grey scale images with inverted colors, while merged channel images are displayed as indicated. Arrowheads denote vesicular cathepsin staining in the peri-nuclear regions, whereas circles indicate immuno-recognized puncta in the extracellular space. Boxed areas in a1-a4, b1-b4, c1-c4, and d1-d4 are shown in higher magnification in a5-a8, b5-b8, c5-c8, and d5-d8, respectively. Note that some primary cilia of human thyroid epithelial cells *in vitro* are positive for cysteine peptidase activity (cyan boxed cilia in **A**), while others lacked proteolytic activity (red boxed cilia in **A**). Cathepsins B and L are abundantly detected in endo-lysosomal vesicles (**B, D**, arrowheads) that are typically located around the base of primary cilia which originate from the centrosomes of the microtubule-organizing centers in juxta-nuclear position. Cathepsin K is more abundant throughout the cytoplasm and present in the extracellular space (**C**, circles). Scale bars represent 20 µm in a1-a4, b1-b4, c1-c4 and d1-d4, while 5 µm are indicated in a5-a8, b5-b8, c5-c8, and d5-d8, respectively.

To analyze whether the cysteine peptidases are proteolytically active at or near primary cilia of human thyroid epithelial cells, Nthy-ori 3–1 cells were used for *in vivo* peptidase activity visualization with the activity-based probe DCG-04 before fixation and immunostaining with anti-ARL13B antibodies. Primary cilia of different lengths were observed in Nthy-ori 3–1 cell cultures and some but not all of them were decorated with active cysteine peptidases (**Figure 9A**). To test whether the most abundant thyroidal cysteine cathepsins B, K, and L are localized at primary cilia, immunostaining was performed with respective antibodies. High-resolution laser scanning microscopy revealed that cathepsins B, K, and L are present in vesicles that surround the cilia base, but proteases are not detected along the cilia (**Figures 9B-D**, respectively). This was particularly obvious for cathepsin L, while cathepsin B- and K-containing structures were additionally found spread throughout the cytoplasm. Additionally, cathepsin K was detected in extracellular puncta (**Figure 9C**).

The results show that cysteine cathepsins B and L are present in juxta-nuclear vesicles of Nthy-ori 3–1 cells, while cathepsin K is also secreted into the extracellular space surrounding the thyrocytes.

### 3.5 Primary cilia appearance upon inhibition of cysteine cathepsins in Nthy-ori 3–1 cells *in vitro*

From the morphological study, we deduced that cathepsin-containing endo-lysosomes are present in the vicinity of the microtubule-organizing center (MTOC; see **Figure 9**), which serves in organizing microtubules for proper formation and elongation of primary cilia (44, 45). We therefore asked whether the frequencies and lengths of primary cilia depend on specific cysteine cathepsin activities in human thyroid epithelial cells. Therefore, Nthy-ori 3–1 cell cultures were incubated for time intervals of 24 h with broad-spectrum and specific cysteine peptidase inhibitors. Namely, the cell-permeable and irreversible broad-spectrum cysteine peptidase inhibitor E64d (also known as Aloxistatin) was used in comparison to specific cysteine cathepsin inhibitors, *i.e.*, CA074me targeting cathepsin B as well as cathepsin L inhibitor III, and Odanacatib, a cathepsin K-specific inhibitor. With this approach we addressed the main thyroglobulin-degrading cysteine cathepsins B, K, and L that are of relevance in human and mouse thyroid tissue (29, 46), except cathepsin S for which no specific inhibitor was available. DMSO treatment served as solvent control.

**Figure 10:**
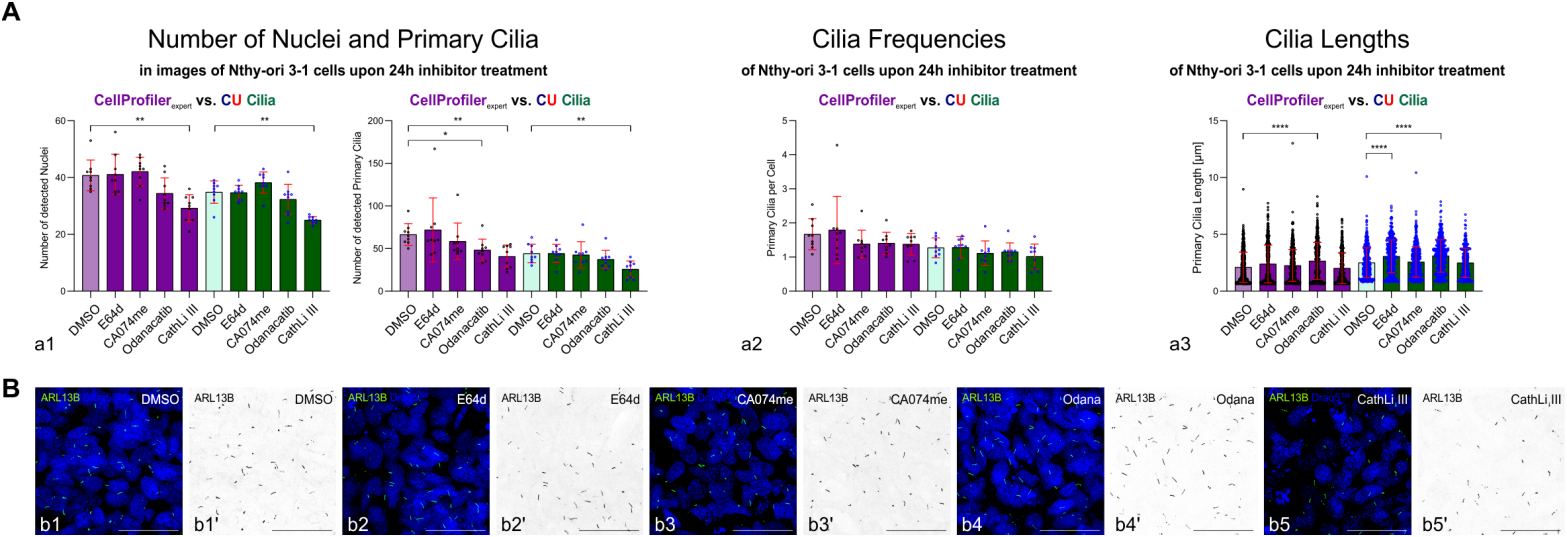
Elongation of primary cilia extending from human thyroid epithelial cells upon treatment with cysteine peptidase inhibitors for 24 h as revealed by CellProfiler™ and CU Cilia analyses. **(A)** Bar charts comparing the numbers of detected nuclei and primary cilia **(a1)**, cilia frequencies **(a2)** and cilia lengths **(a3)** as revealed by CellProfiler™ pipelines set up by an expert user (violet, left panels) and CU Cilia (green, right panels). Identical images (n=9) were analyzed with both approaches. Mean values ± standard deviations are displayed in the bar charts with individual data indicated by circles or dots **(a1–a3)**. Note that both approaches yielded comparable results. Cilia elongation was observed upon treatment with E46d and Odanacatib for 24 h **(a3)**. Statistical analysis was by Kruskal-Wallis and Dunn’s multiple comparisons tests; levels of significance are indicated as * for p<0.01, ** for p<0.001, and **** for p<0.0001. **(B)** Merged channel confocal laser scanning micrographs depicting ARL13B-positive primary cilia (**B**, green in **b1–b5**; black in **b1’–b5’**) and Draq5™-stained nuclei (**B**, blue in **b1–b5**) of DMSO-treated controls **(b1, b1’)** or Nthy-ori 3–1 cell cultures treated with broad-spectrum **(b2, b2’)** or specific inhibitors of cathepsin B **(b3, b3’)**, cathepsin K **(b4, b4’)** or cathepsin L **(b5, b5’)**, respectively. Note that corresponding single channels of anti-ARL13B-positive primary cilia are shown in inverted contrast **(b1’–b5’)** for clarity. Scale bars represent 50 µm. Cathepsin L inhibitor III treatment for 24 h was cytotoxic towards Nthy-ori 3–1 cells as obvious from fewer detected nuclei **(a1)** and more abundant apoptotic bodies **(b5)**.

Image analysis was applied by the expert user’s CellProfiler™ pipeline and by CU Cilia with the aim to determine whether long-term cysteine peptidase inhibitor treatment of confluent Nthy-ori 3–1 cell cultures affects the numbers and lengths of primary cilia (**Figure 10**). Both methods, CU Cilia and CellProfiler™, performed well in detecting nuclei and primary cilia of confluent Nthy-ori 3–1 cell cultures treated for 24 h with the inhibitors. The numbers of detected nuclei and primary cilia were not different among the controls or most of the treatment groups when CU Cilia was used in comparison to the results achieved by the expert user with CellProfiler™ (**Figure 10A, a1**; **Table 5**). However, Nthy-ori 3–1 cells treated with cathepsin L inhibitor III for 24 h were fewer than those in the solvent control group, possibly indicating cytotoxicity, which reached significance in CellProfiler™- and CU Cilia-based analyses (**Figure 10A, a1**; **Table 5**). For further interpretation, it is important to keep in mind that both approaches were effective in identifying nuclei or primary cilia (**Figure 10A, a1**; **Table 5**, **Supplementary Figures 5**, **6**).

**Table 5:** Descriptive statistics of CellProfiler™ *versus* CU Cilia-based analyses of Nthy-ori 3–1 cells treated for 24 h with cysteine peptidase inhibitors in comparison to DMSO solvent controls; the analyses were conducted by an expert user with identical image sets, n=9.

Nuclei counts (see Figure 10A, a1, left)	CellProfiler™ (expert pipeline)					CU Cilia (new parameters)						
Treatment (24 h)	DMSO	E64d	CA074	Odana	CathLi	DMSO		E64d		CA074	Odana	CathLi
Number of values	9	9	9	9	9	9		**9**		**9**	**9**	**9**
Minimum	35	**34**	**32**	**29**	**21**	26		**31**		**30**	**24**	**23**
Maximum	53	**56**	**48**	**44**	**36**	39		**39**		**43**	**42**	**27**
Range	18	**22**	**16**	**15**	**15**	13		**8**		**13**	**18**	**4**
Mean	40.78	**41.11**	**42.11**	**34.44**	**29.22**	34.89		**34.67**		**38.22**	**32.33**	**25**
Std. Deviation	5.38	**7.132**	**5.061**	**5.411**	**4.764**	3.951		**2.598**		**3.768**	**5.315**	**1.225**
Std. Error of Mean	1.793	**2.377**	**1.687**	**1.804**	**1.588**	1.317		**0.866**		**1.256**	**1.772**	**0.4082**
Cilia numbers (see Figure 10A, a1, right)	CellProfiler™ (expert pipeline)					CU Cilia (new parameters)						
Treatment (24 h)	DMSO	E64d	CA074	Odana	CathLi	DMSO		E64d		CA074	Odana	CathLi
Number of values	9	9	9	9	9	9		9		9	9	9
Minimum	50	**43**	**44**	**33**	**22**	30		**22**		**22**	**25**	**13**
Maximum	94	**167**	**113**	**77**	**55**	63		**60**		**78**	**62**	**40**
Range	44	**124**	**69**	**44**	**33**	33		**38**		**56**	**37**	**27**
Mean	66.44	**71.89**	**58.44**	**48.22**	**40.78**	44.22		**44.22**		**42.56**	**37.22**	**25.67**
Std. Deviation	12.74	**37.55**	**21.41**	**12.87**	**13.35**	10.86		**10.65**		**15.45**	**10.72**	**9.76**
Std. Error of Mean	4.246	**12.52**	**7.136**	**4.291**	**4.45**	3.62		**3.55**		**5.151**	**3.574**	**3.253**
Cilia frequencies (see Figure 10A, a2)	CellProfiler™ (expert pipeline)					CU Cilia (new parameters)						
Treatment (24 h)	DMSO	E64d	CA074	Odana	CathLi	DMSO		E64d		CA074	Odana	CathLi
Minimum	1.08	**0.94**	**0.98**	**1**	**0.87**	0.83		**0.61**		**0.55**	**0.88**	**0.54**
Maximum	2.54	**4.28**	**2.35**	**2.08**	**1.77**	1.7		**1.62**		**1.9**	**1.77**	**1.6**
Range	1.46	**3.34**	**1.37**	**1.08**	**0.9**	0.87		**1.01**		**1.35**	**0.89**	**1.06**
Mean	1.67	**1.79**	**1.38**	**1.41**	**1.38**	1.27		**1.28**		**1.11**	**1.15**	**1.02**
Std. Deviation	0.454	**0.989**	**0.406**	**0.323**	**0.316**	0.289		**0.316**		**0.364**	**0.263**	**0.365**
Std. Error of Mean	0.151	**0.33**	**0.135**	**0.108**	**0.105**	0.0964		**0.105**		**0.121**	**0.0876**	**0.122**
Cilia length [µm] (see Figure 10A, a3)	CellProfiler™ (expert pipeline)					CU Cilia (new parameters)						
Treatment (24 h)	DMSO	E64d	CA074	Odana	CathLi	DMSO	E64d		CA074		Odana	CathLi
Number of values	598	**647**	**526**	**434**	**367**	398	**399**		**383**		**335**	**231**
Minimum	0.63	**0.63**	**0.63**	**0.63**	**0.63**	0.79	**0.8**		**0.78**		**0.79**	**0.8**
Maximum	9	**7.7**	**13**	**8.3**	**6.4**	10	**7.5**		**10**		**7.9**	**6.7**
Range	8.3	**7.1**	**12**	**7.7**	**5.7**	9.3	**6.7**		**9.7**		**7.1**	**5.9**
Mean	2.1	**2.4**	**2.3**	**2.7**	**2**	2.5	**3.1**		**2.6**		**3.1**	**2.5**
Std. Deviation	1.3	**1.7**	**1.4**	**1.6**	**1.4**	1.3	**1.4**		**1.3**		**1.4**	**1.3**
Std. Error of Mean	0.054	**0.065**	**0.061**	**0.078**	**0.071**	0.066	**0.072**		**0.069**		**0.078**	**0.084**
Fold-changes	1.00	**1.14**	**1.10**	**1.29**	**0.95**	1.00	**1.24**		**1.04**		**1.24**	**1.00**

Bold means data from inhibitor-treated specimen.

The data for cilia frequencies were not normally distributed for CA074me, E64d and Odanacatib treatments by any of the following tests, *i.e.*, Anderson-Darling, D’Agostino & Pearson, Shapiro-Wilk, and Kolmogorov-Smirnov tests were used. Therefore, statistical analyses were performed by non-parametric Kruskal-Wallis tests for multiple comparisons, which showed that the cilia frequencies did not differ between solvent- or inhibitor-treated cells, no matter, whether CU Cilia or CellProfiler™ was applied (**Figure 10A, a2**). The CellProfiler™ analysis showed an average of 1.79 ± 0.99 cilia per cell in the cells treated with the general cathepsin inhibitor E64d for 24 h, hence, no statistically significant change compared to the DMSO solvent control (1.67 ± 0.45 cilia per cell). Likewise, no difference was recorded in the 24 h specific cysteine cathepsin inhibitor treated cells, namely CA074me (1.38 ± 0.41 cilia per cell), Odanacatib (1.41 ± 0.32 cilia per cell), and cathepsin L inhibitor III (1.38 ± 0.32 cilia per cell), when compared to the DMSO solvent control (**Figure 10A, a2**, pink bars, **Table 5**). Similarly, the **CU Cilia** analysis revealed no difference regarding cilia frequencies between the cells treated for 24 h with E64d (1.28 ± 0.32 cilia per cell), CA074me (1.11 ± 0.36), Odanacatib (1.15 ± 0.26 cilia per cell), and cathepsin L inhibitor III (1.02 ± 0.37 cilia per cell) compared to the DMSO solvent control (1.27 ± 0.29 cilia per cell) (**Figure 10A, a2**, green bars, **Table 5**).

Regarding cilia lengths, however, CellProfiler™ analysis revealed statistically significant differences between Nthy-ori 3–1 cells treated with the cathepsin K-specific inhibitor Odanacatib (2.7 ± 1.6 µm) compared to the DMSO solvent control (2.1 ± 1.3 μm), while general and cathepsin B- or L-specific peptidase inhibitors, namely E64d (2.4 ± 1.7 μm), CA074 (2.3 ± 1.4 μm), and cathepsin L inhibitor III (2.0 ± 1.4 μm) revealed no differences (**Figure 10A, a3**; pink bars, **Table 5**). CU Cilia identified statistically significant differences between the cilia lengths of E64d (3.1 ± 1.4 μm) and Odanacatib (3.1 ± 1.4 μm) treated cells compared to the DMSO solvent control (2.5 ± 1.3 μm), while CA074me (2.6 ± 1.3 μm) and cathepsin L inhibitor III (2.5 ± 1.3 μm) inhibition had no effect on cilia lengths (**Figure 10A, a3**; green bars, **Table 5**). It is noteworthy that the data for primary cilia lengths were not normally distributed by any of the following tests, *i.e.*, Anderson-Darling, D’Agostino & Pearson, Shapiro-Wilk, and Kolmogorov-Smirnov tests were used.

**Figure 11:**
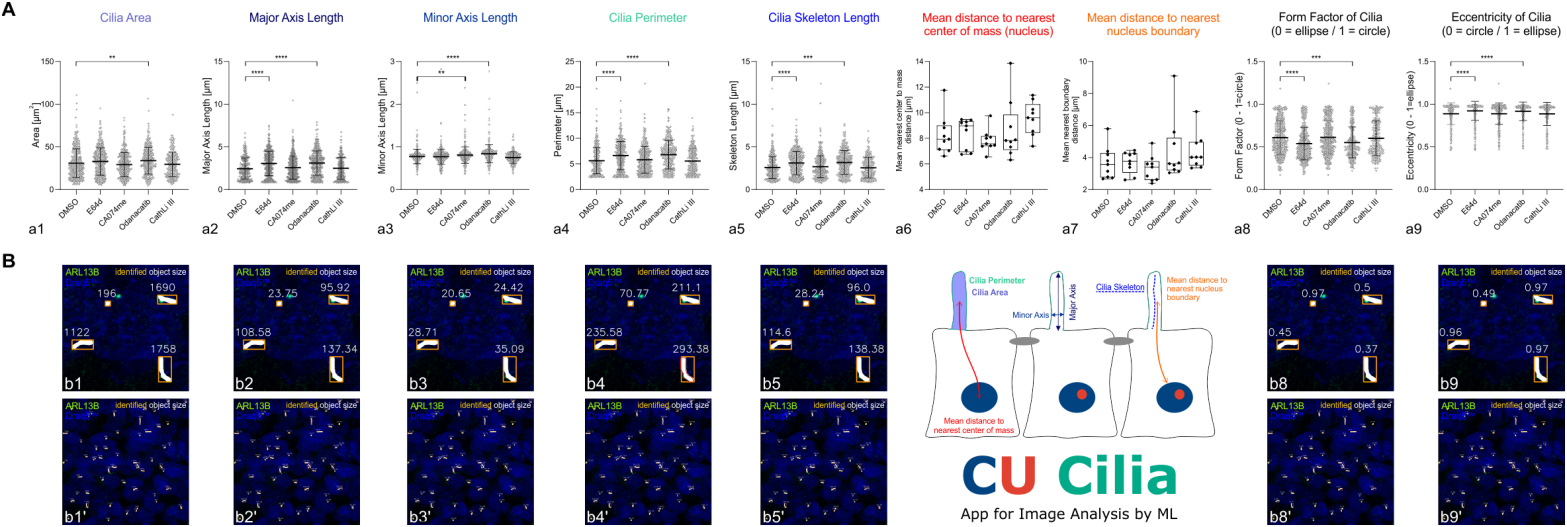
Elongation and thinning of primary cilia extending from human thyroid epithelial cells upon treatment with general cysteine peptidase and cathepsin K-specific inhibitors for 24 h as revealed by advanced CU Cilia analyses. **(A)** Bar charts and box plots comparing the areas of primary cilia **(a1)**, major axis length **(a2)**, minor axis length **(a3)**, cilia perimeters **(a4)**, cilia skeleton lengths **(a5)**, mean distances of cilia to nearest center of mass **(a6)** or to nuclei boundaries **(a7)** as well as form factor **(a8)** or eccentricity of primary cilia **(a9)** as revealed by CU Cilia. Images (n=6) of DMSO-treated controls or Nthy-ori 3–1 cell cultures treated for 24 h with broad-spectrum or specific inhibitors of cathepsin B, cathepsin K, or cathepsin L, respectively, were analyzed and data is denoted as indicated. Mean values ± standard deviations are displayed in the bar charts with individual data indicated by circles or dots **(a1–a5, a8–a9)**, while box plots are displayed in **a6** and **a7**. Statistical analysis was conducted by Kruskal-Wallis and Dunn’s multiple comparisons tests; levels of significance are indicated as ** for p<0.01, *** for p<0.001, and **** for p<0.0001. Cilia elongation **(a2, a4, a5)** and cilia thinning **(a3)** was observed upon treatment with CA074me, E46d and Odanacatib for 24 h, while more elliptic cilia were observed in E64d- and Odanacatib-treated Nthy-ori 3–1 cells, only **(a8, a9)**. **(B)** Output images generated by CU Cilia consisting of merged channel confocal laser scanning micrographs depicting ARL13B-positive primary cilia (**B**, white) and Draq5™-stained nuclei (**B**, blue) with identified objects boxed (**B**, orange) and object sizes indicated in white font **(B)** of Nthy-ori 3–1 control cell cultures. Schematic drawing summarizing the advanced features of CU Cilia measurements.

It can be concluded from the CU Cilia-based cilia length determination that general cysteine peptidase inhibition with E64d and cathepsin K-specific inhibition with Odanacatib resulted in elongation of primary cilia by approx. 24% when a 24 h long-term treatment was applied. This finding correlates well with the notion that cysteine cathepsin activities are important for formation and maintenance of primary cilia in Nthy-ori 3–1 cells. However, the findings were against our previous hypothesis that cathepsin inhibition with the cell-impermeant E64 inhibitor results in cilia shortening (31). Therefore, and because the cilia length distributions did not typically pass the normality tests, we deemed it important to quantitatively describe the primary cilia of Nthy-ori 3–1 cells in further detail by employing the extended features of our CU Cilia App (**Figure 11**).

Using a dataset of six high-resolution images for controls and the respective cysteine peptidase inhibitor treatments for detailed primary cilia structure analysis by CU Cilia revealed no changes in Nthy-ori 3–1 cellular extensions’ areas, except for Odanacatib treatment (**Figure 11A, a1**). Likewise, and as previously observed, the effects of 24 h-treatments with E64d and Odanacatib on cilia lengths were confirmed in this refined image analysis by the parameters major axis length (**Figure 11A, a2**), cilia perimeter (a4), and cilia skeleton length (a5), while also form factor (a8) and eccentricity (a9) indicated significantly longer primary cilia upon inhibition of cysteine peptidases generally and of cathepsin K particularly. The minor axis length (**Figure 11A, a3**) was significantly increased in CA074me and Odanacatib treatments, thus, primary cilia were rendered thinner by inhibition of cathepsin B and K activities, respectively. In contrast, the mean distances to the nearest center of mass, *i.e.*, nuclei centers, and to the nearest nucleus border remained unchanged in any of the inhibitor treatments compared with solvent-treated control cells (**Figure 11A, a6, a7**), which is interpreted as no change in the positioning of primary cilia relative to the cellular position of the nuclei and the juxta-nuclear MTOC.

We conclude that cysteine peptidases, in particular, cathepsin K activities are important for primary cilia length determination in Nthy-ori 3–1 cell cultures *in vitro*. The underlying molecular mechanism, however, remains elusive and requires further studies.

## 4 Discussion

Primary cilia of Nthy-ori 3–1 cells shorten and become less frequent, when this human normal thyroid epithelial cell line is treated with a cell-impermeant broad-spectrum inhibitor of cysteine proteases, *i.e.*, E64 and/or the activity-based probe DCG-04, for extended time periods of up to 20 h (31). This previous study demonstrated that cysteine cathepsins are important to maintain primary cilia of thyroid epithelial cells intact (31), but it remained to be determined which specific cysteine peptidase is responsible for this role in thyroid physiology. In this study, we have approached this question in more detail by analyzing primary cilia extensions in non-treated and thyroid epithelial cells treated with broad-spectrum, cell-permeable and specific cysteine cathepsin inhibitors for short and long time intervals. Fixation and immunostaining protocols for optimized primary cilia preservation and visualization yielded high-resolution images of ARL13B-positive cellular extensions of Nthy-ori 3–1 cells which were evaluated by CU Cilia regarding numerous parameters beyond their frequencies and lengths (see **Figure 11**). We deemed it important to describe primary cilia architecture in detail, because structural alterations or a complete loss of primary cilia are observed in thyroid disorders including cancer (8, 15, 16, 19, 47), indicating that the antennas of thyrocytes can serve as excellent biomarkers of healthy or diseased thyroid tissue. The developed and freely accessible CU Cilia application allows for unbiased primary cilia structure investigations in hundreds of images, making the approach of using primary cilia structural alterations as health and disease biomarkers accessible to scientists of different basic science and medical research fields.

### 4.1 Constructing CU Cilia – state-of-the-art high-content image feature segmentation and challenges

During the recent surge in advancements within deep machine learning techniques for image segmentation over the past few years, numerous innovative approaches have surfaced (36, 48) particularly in the realm of segmenting biological cells, *i.e.*, StarDist (49), YeaZ (50), Cellpose (23), and YeastMate (51) were developed. These methodologies represent a concerted effort to leverage the power of ML models in delineating cell boundaries and characteristics within complex biological images. Notably, tools such as Cellpose have emerged, offering not only robust segmentation algorithms but also intuitive graphical user interfaces (GUIs) for streamlined and adaptable data processing and labeling by humans.

In addition to Cellpose, other notable methodologies such as Cell-ACDC (52) have further extended the limitations of cell segmentation by integrating multiple previous segmentation techniques into a cohesive framework. Moreover, Cell-ACDC goes beyond mere segmentation, offering an array of supplementary features such as GUI functionalities and advanced capabilities like cell tracking and error correction mechanisms. These enhancements not only improve the segmentation process but also streamline downstream analyses and facilitate more accurate interpretation of biological phenomena captured in images. However, despite the considerable progress made in automated cell segmentation, there still exists a gap in methodologies that allow for training of custom models on proprietary or specialized datasets. While tools like Cell-ACDC offer extensive functionalities, they may lack user friendliness required for researchers working with unique biological systems or experimental conditions.

Hence, image analysis tools are routinely used in life sciences, and the scientific community has recently witnessed notable progress in detection methods that are essential to explore the functions of diverse cell types and their constituents. While CellProfiler™ (22) is widely used due to its extensive image analysis features, its demand for manual input slows down analysis, a significant issue in complex experiments where efficiency is key. Recent advancements have introduced tools like CiliaQ (25), detectCilia (26), and ACDC (24), enhancing cilia detection. CiliaQ excels in quantifying cilia from images, but it has a complex interface that challenges non-experts. Likewise, detectCilia requires knowledge in R, while ACDC offers a strong detection framework but also requires considerable manual tuning, demanding time, and expertise.

Addressing the above outlined limitations represents a crucial frontier in the field, where future advancements may focus on developing adaptable frameworks that empower researchers to train and deploy custom segmentation models tailored to their specific imaging requirements. We have achieved this goal by describing the CU Cilia tool herein.

### 4.2 CU Cilia benchmarking

Although we initially thought that it must be easy to detect primary cilia and segment those from non-stained background (because antibodies against cilia components are excellent and highly specific for primary cilia), it turned out that segmenting the cellular antennas was a challenge. This is probably because of the broad variety of differently shaped primary cilia with wide-ranging length distribution and bent appearances (see **Figure 3**). Importantly, it was not predictable which primary cilia would be identified better by either image analysis tool used in this study, CellProfiler™ or CU Cilia. Therefore, we included visualization of the results to be able judging on which of the approaches, or both, identify cellular antennas best or better (see **Figure 7**). It was further important to determine the primary cilia frequencies and therefore cell counting was essential, specifically in dense cultures of Nthy-ori 3–1 cells that mimic the *in situ* conditions of thyroid epithelial cells being present in a confluent, spherical monolayer around the thyroid follicle lumen. We started our approach by using CellProfiler™ as before (31) and another well-established image analysis tool, Cellpose, to segment the nuclei. When comparing the results achieved by CellProfiler™ and Cellpose, it became clear that the latter was more reliable because here, the edge detection within images is based on training sets using expert labeled images, which do not strictly require a high signal-to-noise ratio. We conclude that primary cilia of “difficult” images are segmented by both, CellProfiler™ and CU Cilia, with high precision and to well-comparable extents, thus, underlining reliability and effectiveness of our newly developed CU Cilia App in image analysis of delicate cellular structures affected by different experimental scenarios like, *e.g.*, protease inhibitor treatments.

In conclusion, the above experimental results confirmed our hypothesis that it is enough to fine-tune the nuclei segmentation model of CU Cilia on non-treated thyroid epithelial cell datasets (train set A) to yield high quality segmentation results also for treated thyroid epithelial cell datasets (train set B), while also outperforming the original Cellpose method. In addition, we have shown that fine-tuning the model on only a small amount of data gives high-quality results which are only slightly worse than for a model trained on the entire dataset. The latter observation explains why CU Cilia is easier to use than Cellpose. Namely, it allows us to spend less effort on labeling new image datasets by expert users. In other words, CU Cilia can be used without any fine-tuning or pre-training, and it will still yield excellent results on thyroid epithelial cell nuclei segmentation.

CU Cilia goes beyond CellProfiler™ and Cellpose with its easy-to-train and fast-to-run approach with the following implementations:

- Thyrocyte image datasets, manually labeled for nuclei segmentation and cilia detection, were used for ML algorithms’ training; this CU Cilia dataset is publicly available (see link in https://constructor.app/platform/research/public/project/cu_cilia).
- We fine-tuned the Cellpose method on this dataset, which allowed to significantly improve the quality of nuclei segmentation. We also demonstrated the possibility of effective fine-tuning this segmentation ML model, namely, by using only a small amount of data, *i.e.*, few images.
- We developed a new cilia detection approach that is equal to or superior to CellProfiler™ in speed and accuracy of describing the architecture of the delicate cellular antennas.
- The nuclei segmentation and cilia detection methods formed the basis of the CU Cilia App pipeline, which analyzes input images of immuno-stained cells, and computes characteristics that allow us to describe the nuclei and cilia of the input images. It is important to note that the CU Cilia App has both a low entry threshold and optional flexible settings. The CU Cilia App is deployed ready to use on the Constructor Platform.
- The practical value and ease of use of CU Cilia were confirmed by us through research that extended our previous investigations on the significance of cysteine peptidases -with important functions in thyroid physiology- for primary cilia formation and maintenance of their architecture.

With the CU Cilia App in use, researchers can now easily test drug-induced effects toward thyroid cells. In the long run, CU Cilia is meant to diagnose thyroid disorders in an automated, fast, highly reproducible, and objective fashion. Most importantly, there is no need for lengthy instructions or practice sessions, because CU Cilia is made such that it can be used intuitively by non-experts and expert researchers alike. Another important aspect is that CU Cilia is implemented in the Constructor Platform which provides computing resources, a fully configured hardware and software execution environment, storage space from 50 Gb to 2000 Gb depending on the user plan, and the ready-to-run CU Cilia application.

An important advantage of this approach is that the user only needs to upload the respective image dataset to the platform, to indicate in the application settings the folder which contains this dataset, and then click the Start-button to receive the results within minutes, typically less than one second is needed per image. For the more advanced user, however, it is also possible to adapt the basic parameters of CU Cilia, if deemed necessary. The CU Cilia on the Constructor Platform can be found at the following link (https://constructor.app/platform/research/public/project/cu_cilia). The source code is also provided in GitHub (https://github.com/andrewbo29/cu_cilia).

**Figure 12:**
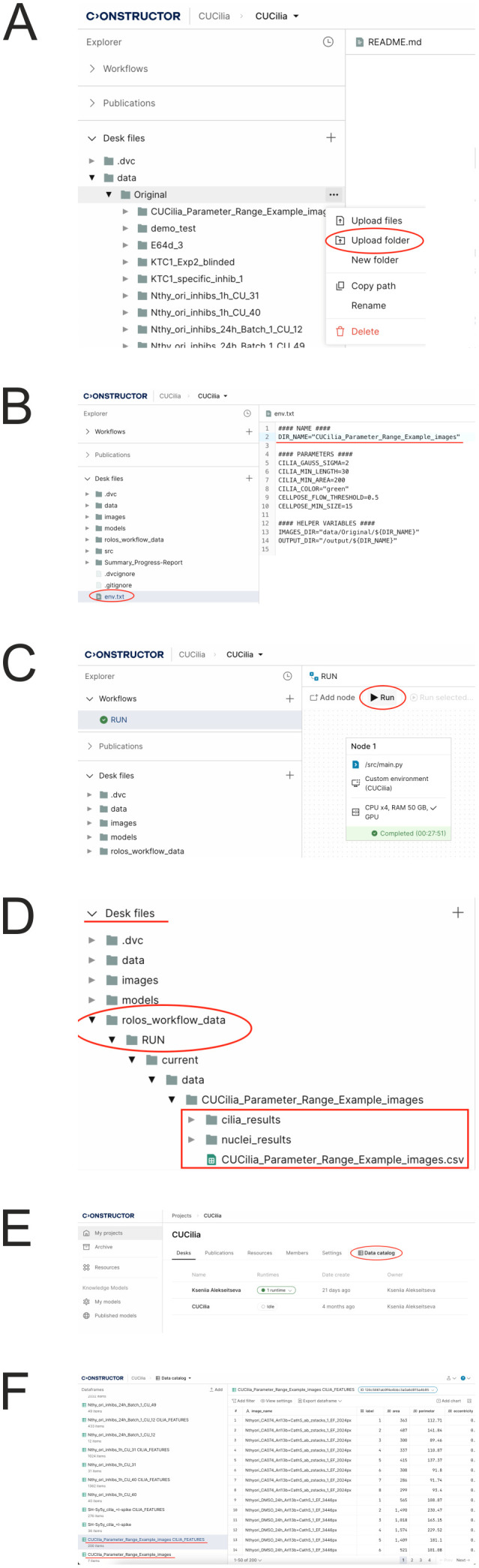
Step-by-step instructions for running CU Cilia on the Constructor Platform (CP). **(A)** Inside the ‘data/Original’ folder (create it if it does not exist), create a new folder and upload your images there. Only ‘.tiff’ and ‘.tif’ image formats are supported. A nested folder structure is also possible if necessary. **(B)** In ‘env.txt`, change the ‘DIR_NAME’ to the name of the folder you created (*e.g.*, ‘CUCilia_Parameter_Range_Example_images’) and modify other parameters (descriptions are given in **Table 1**) if needed. **(C)** Navigate to the ‘Workflows’ section, select the ‘RUN’ workflow, and click the ‘▶ Run’ button to start processing. **(D)** After the workflow is complete, you can find the results (images with detected cilia and nuclei, as well as CSV files with aggregated results) in the ‘rolos_workflow_data/RUN’ folder within ‘Desk’ files. **(E, F)** The results will be available in the ‘Data Catalog’ in the main project window, which includes two tables: one detailing the characteristics of each cilium (table with ‘CILIA_FEATURES’ suffix) and another providing aggregated measurements for each image.

In essence, CU Cilia detects primary cilia with the same precision as CellProfiler™, while CU Cilia detects nuclei with higher precision than CellProfiler™. Overall, CU Cilia is easier to use by a non-expert, because the image analysis parameters are predefined, while setting up a CellProfiler™ pipeline may be very versatile but requires expert knowledge and considerable time. Moreover, for both expert and non-expert image analysis users, CU Cilia outperforms CellProfiler™ in processing speed and ease of use. Enhanced automation significantly reduces processing time, even for large batches of data, making it an efficient solution for cilia and nuclei segmentation. This efficiency is particularly advantageous in high-throughput settings where rapid and accurate analysis is paramount. CU Cilia also demonstrates high-quality performance on new, unseen data, underscoring its robustness and generalizability. To further detail the ease of use of CU Cilia, step-by-step instructions for running CU Cilia on the Constructor Platform (CP) are provided (**Figure 12**).

In summary, CU Cilia should not only match but may also surpass the performance of other image analysis tools, mainly by offering additional capabilities and user-friendly features.

### 4.3 Back to the biology - primary cilia of thyroid epithelial cells – possible biological implications as sensory biomarkers, antennas in drug validation studies, and disease forecasting structures

Thyroid hormones (TH) are essential for almost all cells of the body in all phases throughout life. Hence, the thyroid gland carries vital functions in providing peripheral organs and the central nervous system with enough TH. This is achieved by a fine-tuned balance of thyroglobulin biosynthesis, storage in the thyroid follicle lumen, and by its proteolytic processing for liberation of TH. The proteases important for TH liberation belong to the cysteine cathepsins, which can act on thyroglobulin while it is still in the thyroid follicle lumen, and they will continue its processing and degradation within endo-lysosomes upon re-internalization of thyroglobulin (13, 14). Thus, there must be a sensing mechanism that controls the status of thyroglobulin within the follicle lumen. Primary cilia are well known to act as mechanosensory antennas with functions in probing the pericellular environment of cells, but this task has not yet been described for within thyroid follicles.

Primary cilia extend from the apical surface of thyroid epithelial cells towards the lumen of thyroglobulin-containing follicles (13, 53). Accordingly, primary cilia of thyrocytes have been related to TSH-regulated thyroglobulin re-internalization from within the thyroid follicle lumen (15). Interestingly, primary cilia at the apical pole of rodent thyroid epithelial cells harbor another G protein-coupled receptor besides the basolateral TSH receptor that is involved in the regulation of thyroid physiology, namely, the trace amine-associated receptor 1 (Taar1) (33, 53, 54). However, whether Taar1/TAAR1 has any role in rodent or human primary cilia functions is not yet understood. One image dataset used in this study was from Nthy-ori 3–1 cells that were transduced to stably expressed this G protein-coupled receptor (33). The data showed that both easy and difficult to detect primary cilia, with features well-comparable to those cilia of solvent control cells, were present in such cell preparations (see **Tables 3**, **4**). This leads us to the conclusion that TAAR1 expression is not a main determinator of cilia architecture. Hence, the precise roles of G protein-coupled receptors of primary cilia in thyroid physiology remain open and it is unclear whether TH derivatives, like 3-iodothyronamine, might be involved in mitigating primary cilia functions in the healthy thyroid gland (53, 54). Still, the localization of TSH-triggered, secreted and cell surface re-associated proteases at or near primary cilia is intriguing, because they are critical for TH liberation by proteolytic processing of thyroglobulin (29–31, 46, 55). Therefore, we suggested the involvement of primary cilia in sensing/probing the molecular composition of the colloid in the thyroid follicle lumen (13).

Our previous study (31) showed that cysteine peptidase inhibition with cell-impermeable E64 and DCG-04 resulted in shortening of primary cilia, whereas this study demonstrates that the use of cell-permeable E64d results in elongation of primary cilia. We conclude that it indeed matters whether cysteine peptidases are broad-spectrum inhibited in the extracellular space and within the endocytic compartments (31) or alternatively, whether cytosolic cysteine peptidases are inhibited as well (this study).

Importantly, however, the results of this study suggest that primary cilia elongate when cathepsin K activity is inhibited. This notion is interesting because the cathepsin K-specific small molecule inhibitor Odanacatib was extensively studied as an anti-osteoporotic drug, hence, it was thought to be used in preventing excessive cathepsin K-mediated bone resorption. However, the clinical trials were halted due to enhanced risk of stroke in patients treated with Odanacatib (56–59). After a long period of time, cathepsin K inhibitors are now re-assessed in osteoclast-mediated bone resorption (60) and re-evaluated for treatment of bone disease caused by metastasizing (breast) carcinoma cells (61, 62).

Interestingly, the data of this study, namely, indicating elongation and structural changes of primary cilia upon cathepsin K inhibition, correlates well with a recent study on osteoclasts (63), which express high amounts of cathepsin K-enabling bone resorption. Formation of osteoclasts from their hematopoietic precursor cells -macrophages- requires disassembly of primary cilia, while their pharmacologically induced elongation results in down-regulation of cathepsin K expression (63). Taking together with the results of this study, we deduce that cathepsin K is indeed important for regulation of primary cilia length, not only in thyrocytes but possibly also in other cell types like osteoclasts. Further investigations in that regard are underway.

In conclusion, the advanced CU Cilia tool for detection of cilia and their analysis in microscopy images provides enhanced automation and efficiency, enabling rapid and accurate analysis. We propose that the ability to analyze cilia rapidly without the need to fine-tune detection parameters and calculate comprehensive features will position CU Cilia as a superior choice for researchers and practitioners in the field alike.

## Data Availability

The datasets presented in this study can be found in online repositories. The names of the repository/repositories and accession number(s) can be found in the article/**Supplementary Material**.
