# Supplementary material for: CU Cilia – an application for image analysis by machine learning – reveals significance of cysteine cathepsin K activity for primary cilia of human thyroid epithelial cells
Source: Front Endocrinol (Lausanne). 2025 Nov 27;16:1588394. doi: 10.3389/fendo.2025.1588394 (PMC12695601; doi:10.3389/fendo.2025.1588394)
Supplement: Supplementary file 1 [file DataSheet1.pdf]

Supplementary Material

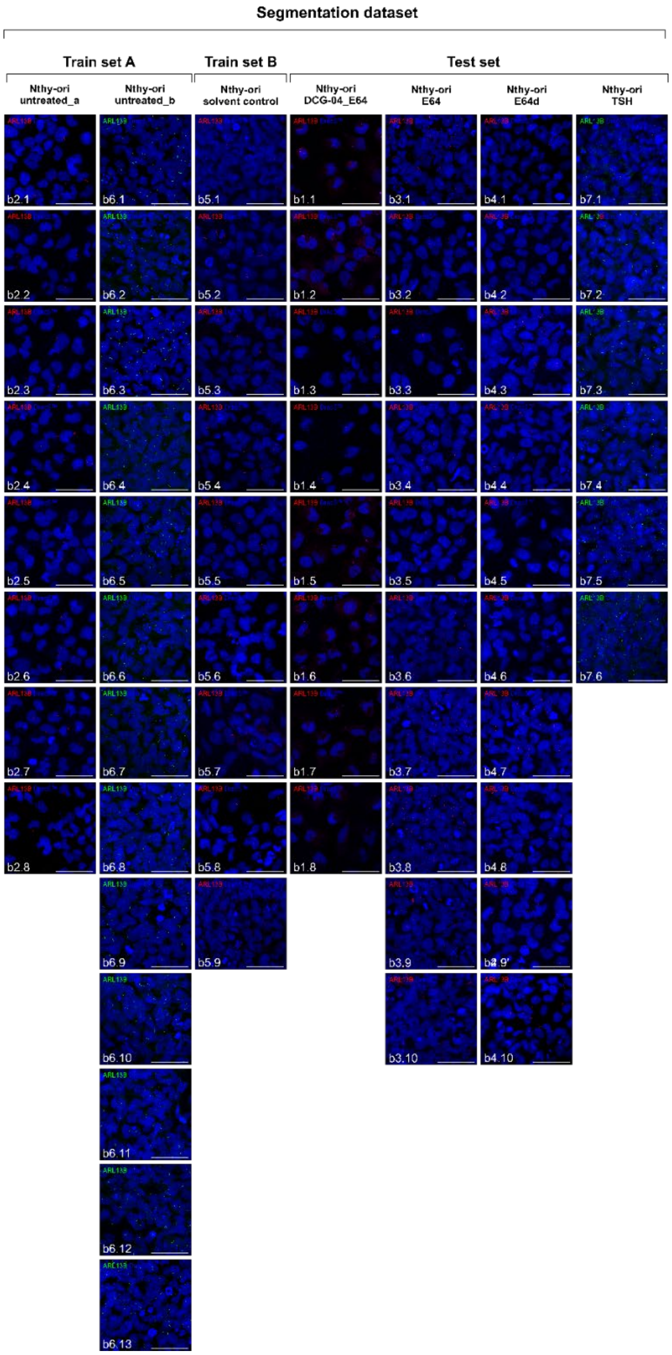

Supplementary Figure 1: Segmentation dataset, consisting of non-treated cells (train set A) and treated cells (train set B), i.e., "Nthy-ori_untreated_a image 1-8", "Nthy-ori_untreated_b image 1-13", "Nthy-ori_solvent_ctrl image 1-9". Test set, consisting of "Nthy-ori_DCG04_E64 image 1-8", "Nthy-ori_E64 image 1-10", "Nthy-ori_inhib_E64d image 1-10", and "Nthy-ori_TSH image 1-6"**.** Scale bars represent 50 µm.

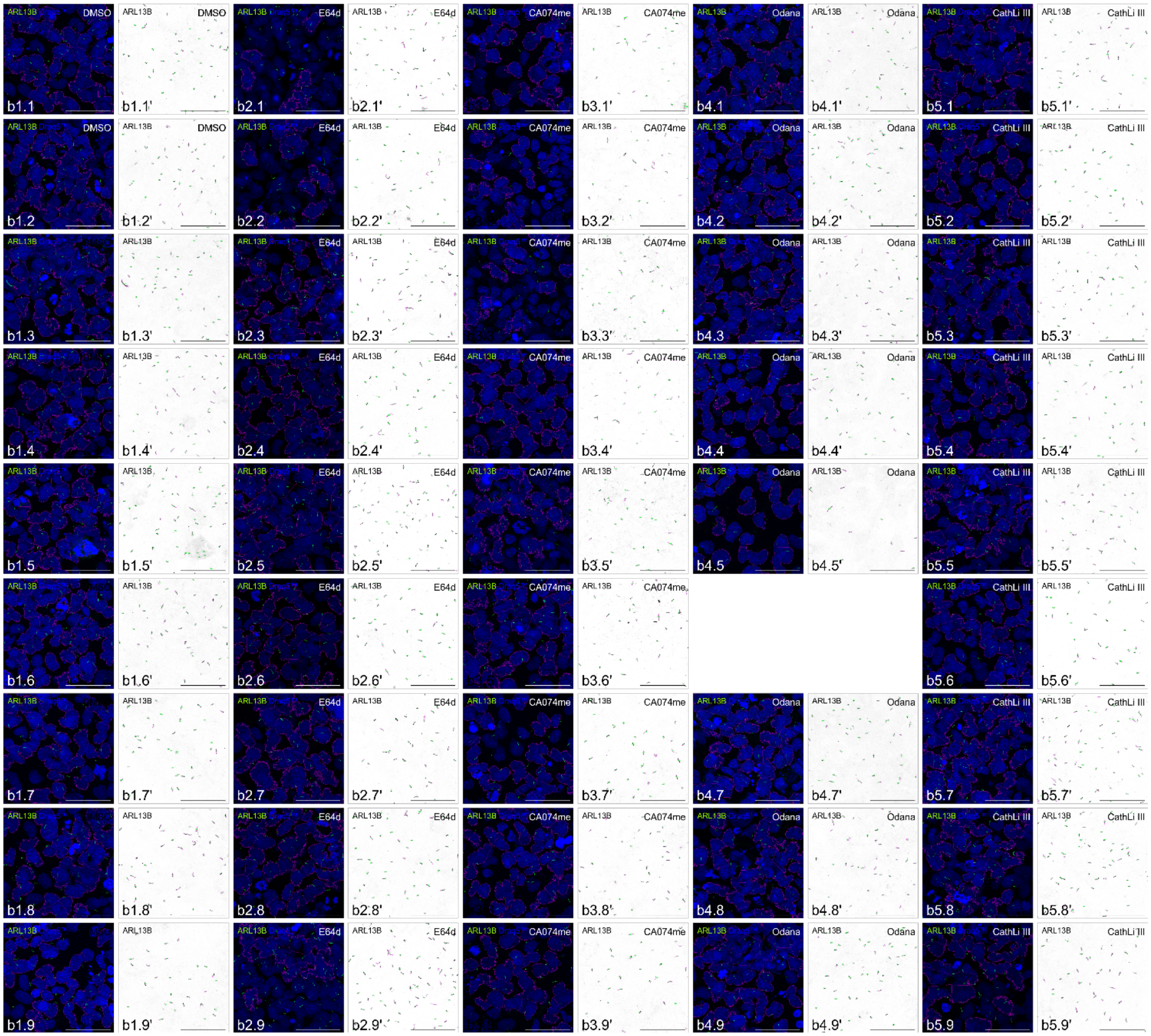

Supplementary Figure 2: Images of 1 h treated cells analyzed by CellProfiler™ pipeline "non-expert". Merged channel confocal laser scanning micrographs depicting ARL13B-positive primary cilia (green in b1.1-b5.9; black in b1.1'-b5.9') and Draq5™-stained nuclei (blue in b1.1-b5.9) of DMSO-treated controls (b1.1-b1.9) or Nthy-ori 3-1 cell cultures treated with broad-spectrum (b2.1-b2.9) or specific inhibitors of cathepsin B (b3.1-b3.9), cathepsin K (b4.1-b4.9) or cathepsin L (b5.1-b5.9), respectively. Note that corresponding single channels of anti-ARL13B-positive primary cilia are shown in inverted contrast (b1.1'-b5.9') for clarity. Identified nuclei and cilia are outlined by red lines (b1.1-b5.9) and red and cyan boxes (b1.1'-b5.9'), respectively. Scale bars represent 50 µm.

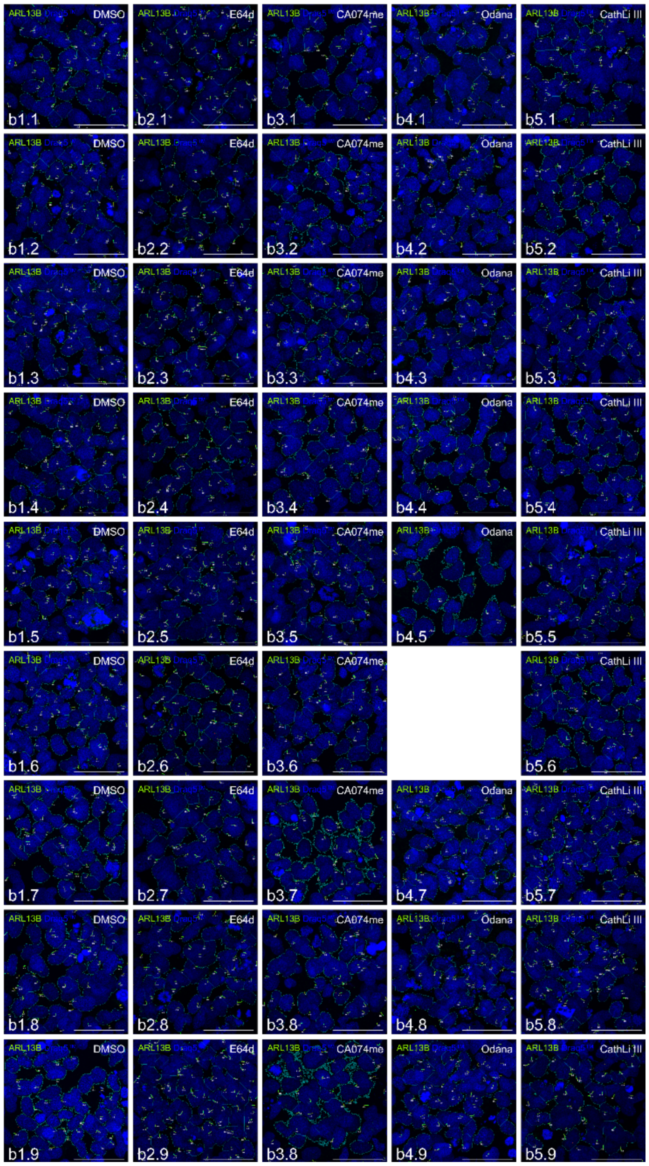

Supplementary Figure 3: Images of 1 h treated cells analyzed by CellProfiler™ pipeline "expert". Merged channel confocal laser scanning micrographs depicting ARL13B-positive primary cilia (green) and Draq5™-stained nuclei (blue) of DMSO-treated controls (b1.1-b1.9) or Nthy-ori 3-1 cell cultures treated with broad-spectrum (b2.1-b2.9) or specific inhibitors of cathepsin B (b3.1-b3.9), cathepsin K (b4.1-b4.9) or cathepsin L (b5.1-b5.9), respectively. Identified nuclei are outlined by green lines and primary cilia by yellow lines (b1.1-b5.9), respectively. Scale bars represent 50 µm.

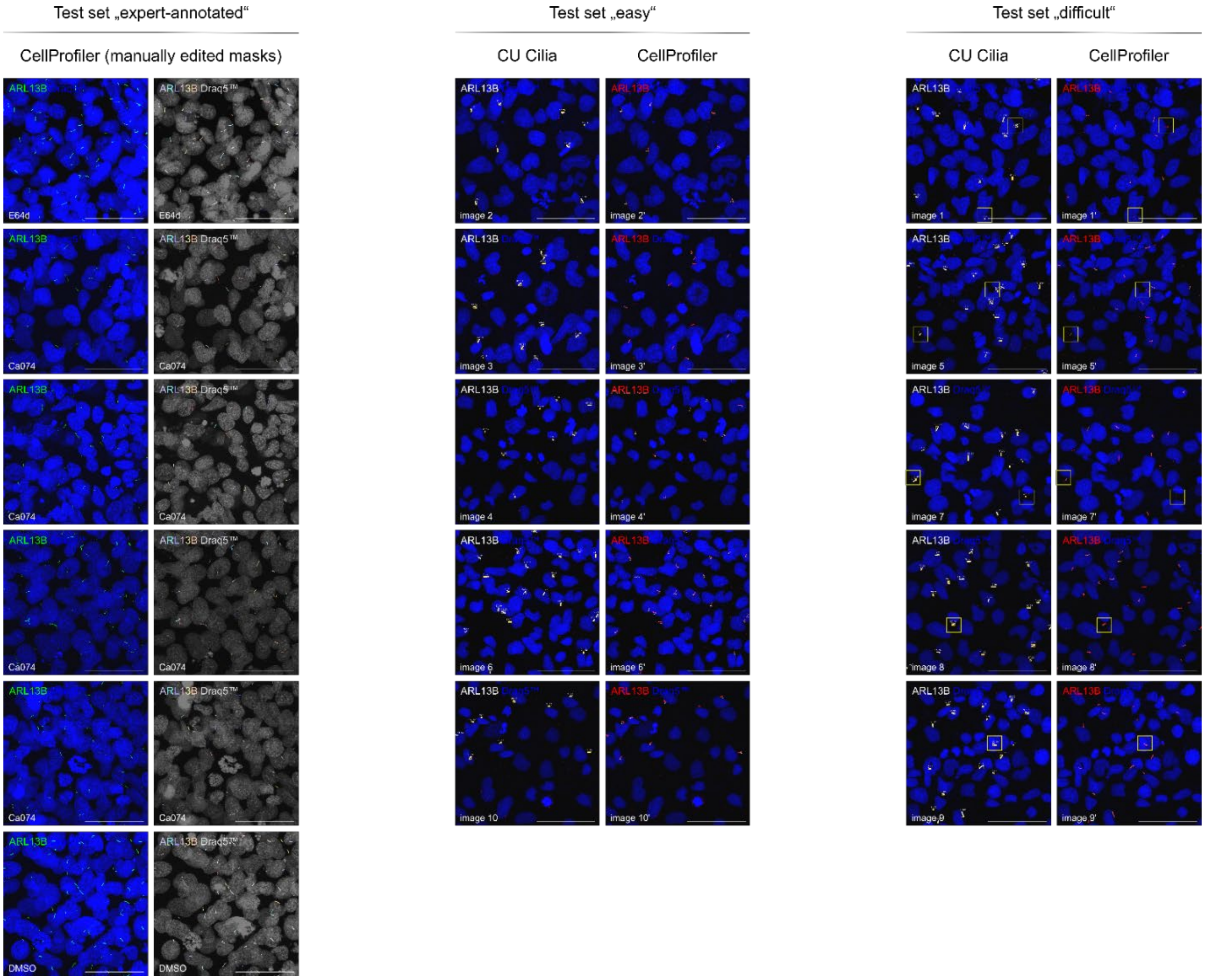

Supplementary Figure 4: Export-annotated data, “easy” cases and “difficult” cases. Expert-annotated images of differently treated cells and images of the test set used for comparison of cilia detection results using CellProfiler™ or CU Cilia, as indicated. Identified primary cilia are outlined by lines or boxes, respectively. Scale bars represent 50 µm.

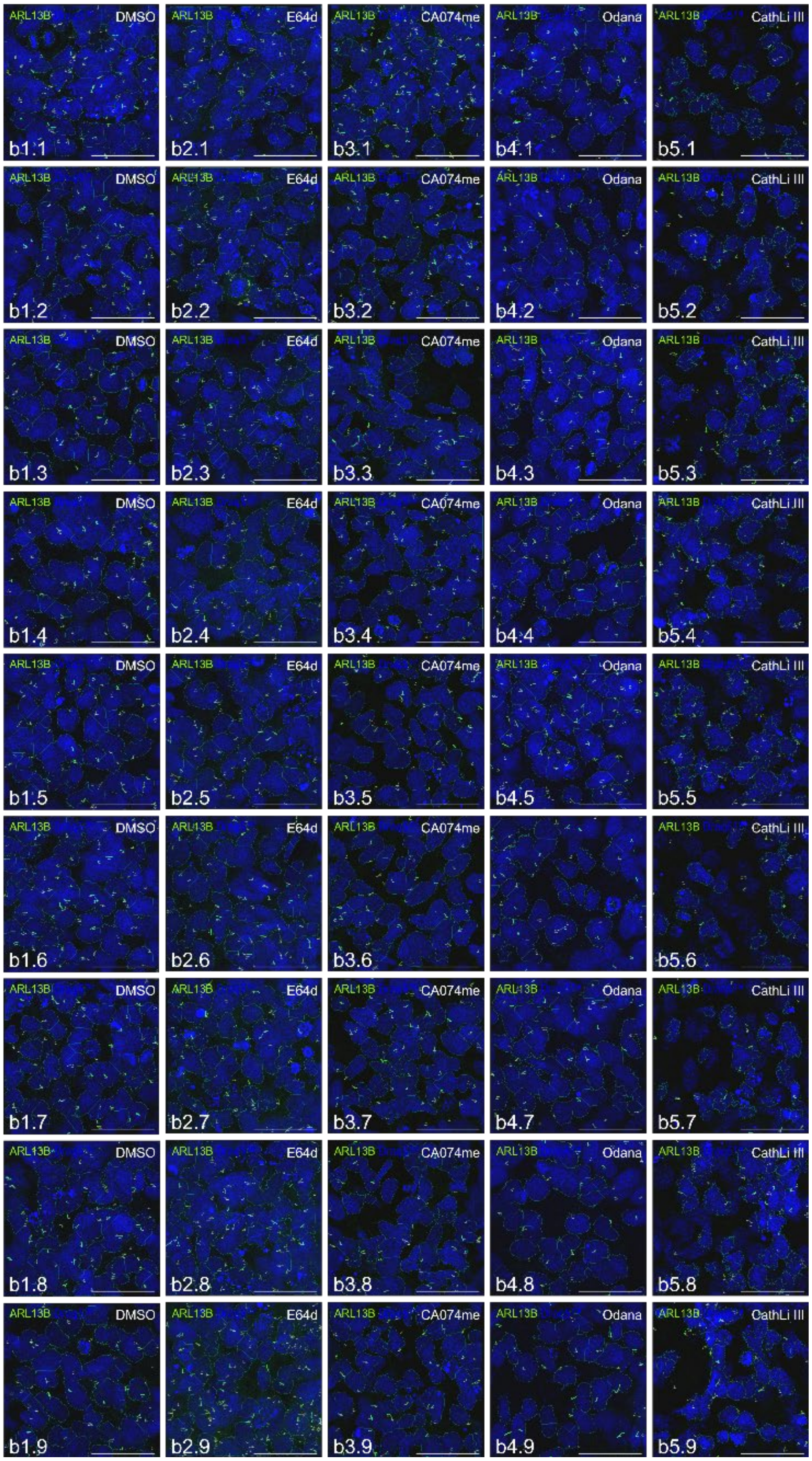

Supplementary Figure 5: Images of 24 h treated cells analyzed by CellProfiler™ pipeline "expert". Merged channel confocal laser scanning micrographs depicting ARL13B-positive primary cilia (green) and Draq5™-stained nuclei (blue) of DMSO-treated controls (b1.1-b1.9) or Nthy-ori 3-1 cell cultures treated with broad-spectrum (b2.1-b2.9) or specific inhibitors of cathepsin B (b3.1-b3.9), cathepsin K (b4.1-b4.9) or cathepsin L (b5.1-b5.9), respectively. Identified nuclei are outlined by green lines and primary cilia by yellow lines (b1.1-b5.9), respectively. Scale bars represent 50 µm.

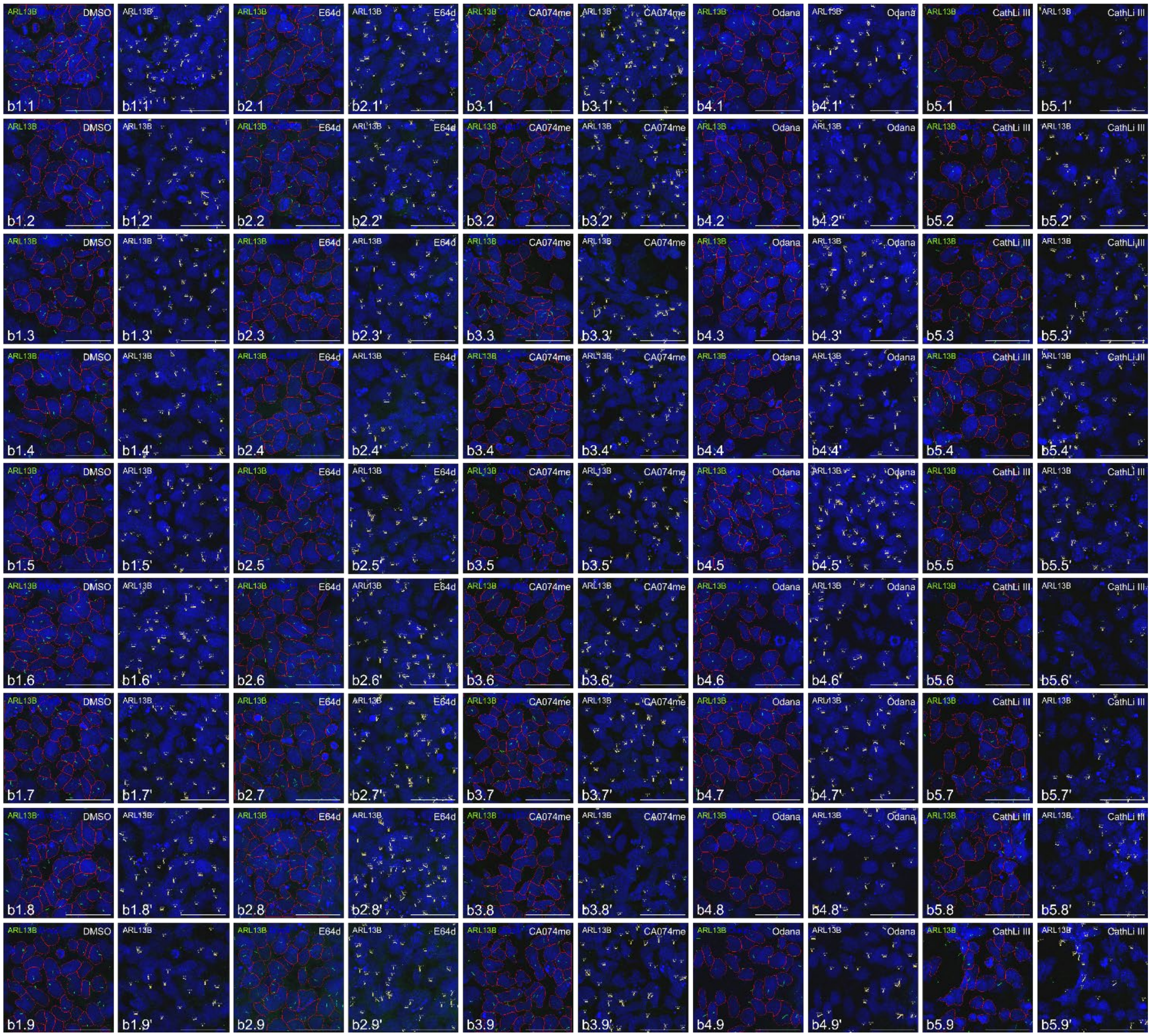

Supplementary Figure 6: Images of 24 h treated cells analyzed by CU Cilia. Merged channel confocal laser scanning micrographs depicting ARL13B-positive primary cilia (green in b1.1-b5.9; white in b1.1’-b5.9’) and Draq5™-stained nuclei (blue in b1.1-b5.9’) of DMSO-treated controls (b1.1-b1.9’) or Nthy-ori 3-1 cell cultures treated with broad-spectrum (b2.1-b2.9’) or specific inhibitors of cathepsin B (b3.1-b3.9’), cathepsin K (b4.1-b4.9’) or cathepsin L (b5.1-b5.9’), respectively. Identified nuclei are outlined by red lines (b1.1-b5.9) and primary cilia by white and yellow boxes (b1.1’-b5.9’), respectively. Scale bars represent 50 µm.

Supplementary Table 1: CellProfiler™ Pipeline Settings “expert user”.

<table>
<tr><td>CellProfiler_Version: 4.2.6</td></tr>
<tr><td>ChannelType_original : Color</td></tr>
<tr><td>ImageSet_Zip_Dictionary</td></tr>
<tr><td align="center">Pipeline_Pipeline (CellProfiler Pipeline: http://www.cellprofiler.org)</td></tr>
<tr><td>Version:5</td></tr>
<tr><td>DateRevision:426</td></tr>
<tr><td>ModuleCount:21</td></tr>
<tr><td>HasImagePlaneDetails:False</td></tr>
<tr><td>Images:[module_num:1|svn_version:'Unknown'|variable_revision_number:2|show_window:False|notes:['To begin creating your project</td></tr>
<tr><td>Filter images?:Images only</td></tr>
<tr><td>Select the rule criteria: and (extension does isimage) (directory doesnot containregexp ""[\\\\\\\\/]\\\\."")</td></tr>
<tr><td>Metadata:[module_num:2|svn_version:'Unknown'|variable_revision_number:6|show_window:False|notes:['The Metadata module optionally allows you to extract information describing your images (i.e metadata) which will be stored along with your measurements. This information can be contained in the file name and/or location</td></tr>
<tr><td>Extract metadata?:No</td></tr>
<tr><td>Metadata data type:Text</td></tr>
<tr><td>Metadata types:{}</td></tr>
<tr><td>Extraction method count:1</td></tr>
<tr><td>Metadata extraction method: Extract from image file headers</td></tr>
<tr><td>Metadata source:File name</td></tr>
<tr><td>Regular expression to extract from file name:^(?P<Plate>.*)_(?P<Well>[A-P][0-9]{2})_s(?P<Site>[0-9])_w(?P<ChannelNumber>[0-9])</td></tr>
<tr><td>Regular expression to extract from folder name:(?P<Date>[0-9]{4}_[0-9]{2}_[0-9]{2})$</td></tr>
<tr><td>Extract metadata from: All images</td></tr>
<tr><td>Select the filtering criteria: and (file does contain """")</td></tr>
<tr><td>Metadata file location:Elsewhere...|</td></tr>
<tr><td>Match file and image metadata:[]</td></tr>
<tr><td>Use case insensitive matching?:No</td></tr>
<tr><td>Metadata file name:</td></tr>
<tr><td>Does cached metadata exist?:No</td></tr>
<tr><td>NamesAndTypes:[module_num:3|svn_version:'Unknown'|variable_revision_number:8|show_window:False|notes:['The NamesAndTypes module allows you to assign a meaningful name to each image by which other modules will refer to it.']|batch_state:array([] dtype=uint8)|enabled: True|wants_pause:False]</td></tr>
<tr><td>Assign a name to: All images</td></tr>
<tr><td>Select the image type: Color image</td></tr>
<tr><td>Name to assign these images: original</td></tr>
<tr><td>Match metadata:[]</td></tr>
<tr><td>Image set matching method: Order</td></tr>
<tr><td>Set intensity range from: Image metadata</td></tr>
<tr><td>Assignments count:3</td></tr>
<tr><td>Single images count:0</td></tr>
<tr><td>Maximum intensity:255.0</td></tr>
<tr><td>Process as 3D?:No</td></tr>
<tr><td>Relative pixel spacing in X:1.0</td></tr>
<tr><td>Relative pixel spacing in Y:1.0</td></tr>
<tr><td>Relative pixel spacing in Z:1.0</td></tr>
<tr><td>Select the rule criteria: and (file does contain ""D.TIF"")</td></tr>
<tr><td>Name to assign these images: Original</td></tr>
<tr><td>Name to assign these objects: Cell</td></tr>
<tr><td>Select the image type: Grayscale image</td></tr>
<tr><td>Set intensity range from: Image metadata</td></tr>
</table>

Maximum intensity:255.0
Select the rule criteria:and (file does contain ""F.TIF"")
Name to assign these images: OrigGreen
Name to assign these objects: Nucleus
Select the image type: Grayscale image
Set intensity range from: Image metadata
Maximum intensity:255.0
Select the rule criteria: and (file does contain ""R.TIF"")
Name to assign these images: OrigRed
Name to assign these objects: Cytoplasm
Select the image type: Grayscale image
Set intensity range from: Image metadata
Maximum intensity:255.0
Groups:[module_num:4|svn_version:'Unknown'|variable_revision_number:2|show_window:False|notes:['The Groups module optionally allows you to split your list of images into image subsets (groups) which will be processed independently of each other. Examples of groupings include screening batches, microtiter plates, time-lapse movies
Do you want to group your images?:No
grouping metadata count:1
Metadata category:None
ColorToGray:[module_num:5|svn_version:'Unknown'|variable_revision_number:4|show_window:False|notes:[]|batch_state:array([] dtype=uint8)|enabled: True|wants_pause:False]
Select the input image: original
Conversion method:Split
Image type:RGB
Name the output image: OrigGray
Relative weight of the red channel:1.0
Relative weight of the green channel:1.0
Relative weight of the blue channel:1.0
Convert red to gray?:No
Name the output image: OrigRed
Convert green to gray?:Yes
Name the output image: OrigGreen
Convert blue to gray?:Yes
Name the output image: OrigBlue
Convert hue to gray?:Yes
Name the output image: OrigHue
Convert saturation to gray?:Yes
Name the output image: OrigSaturation
Convert value to gray?:Yes
Name the output image: OrigValue
Channel count:1
Channel number:1
Relative weight of the channel:1.0
Image name:Channel1
ColorToGray:[module_num:6|svn_version:'Unknown'|variable_revision_number:4|show_window:False|notes:[]|batch_state:array([] dtype=uint8)|enabled:True|wants_pause:False]
Select the input image: original
Conversion method:Combine
Image type:RGB
Name the output image: OrigBlueRed
Relative weight of the red channel:1.0
Relative weight of the green channel:0

Relative weight of the blue channel:1.0
Convert red to gray?:Yes
Name the output image: OrigRed
Convert green to gray?:Yes
Name the output image: OrigGreen
Convert blue to gray?:Yes
Name the output image: OrigBlue
Convert hue to gray?:Yes
Name the output image: OrigHue
Convert saturation to gray?:Yes
Name the output image: OrigSaturation
Convert value to gray?:Yes
Name the output image: OrigValue
Channel count:1
Channel number:1
Relative weight of the channel:1.0
Image name:Channel1
RescaleIntensity:[module_num:7|svn_version:'Unknown'|variable_revision_number:3|show_window:False|notes:[]|batch_state:array([] dtype=uint8)|enabled:True|wants_pause:False]
Select the input image: OrigGreen
Name the output image: RescaledGreen
Rescaling method: Choose specific values to be reset to a custom range
Method to calculate the minimum intensity: Custom
Method to calculate the maximum intensity: Custom
Lower intensity limit for the input image: 0.0
Upper intensity limit for the input image: 1.0
Intensity range for the input image: 0.08 – 1.0
Intensity range for the output image: 0.0 -1.0
Select image to match in maximum intensity: None
Divisor value:1.0
Divisor measurement:None
EnhanceOrSuppressFeatures:[module_num:8|svn_version:'Unknown'|variable_revision_number:7|show_window:False|notes:[]|batch_state:array([] dtype=uint8)|enabled:True|wants_pause:False]
Select the input image: RescaledGreen
Name the output image: EnhancedGreen
Select the operation:Enhance
Feature size:20
Feature type:Neurites
Range of hole sizes: 1 -10
Smoothing scale: 2.0
Shear angle:0.0
Decay:0.95
Enhancement method:Line structures
Speed and accuracy:Fast
Rescale result image:Yes
IdentifyPrimaryObjects:[module_num:9|svn_version:'Unknown'|variable_revision_number:15|show_window:True|notes:['Identify the nuclei from the DAPI image. |batch_state:array([] dtype=uint8)|enabled:True|wants_pause:False]
Select the input image: OrigBlue
Name the primary objects to be identified: Nuclei
Typical diameter of objects in pixel units (Min Max): 180 -750
Discard objects outside the diameter range?:Yes
Discard objects touching the border of the image?:Yes
Method to distinguish clumped objects: Intensity

Method to draw dividing lines between clumped objects: Propagate
Size of smoothing filter:130
Suppress local maxima that are closer than this minimum allowed distance:150
Speed up by using lower-resolution image to find local maxima?:Yes
Fill holes in identified objects?: After declumping only
Automatically calculate size of smoothing filter for declumping?: No
Automatically calculate minimum allowed distance between local maxima?: No
Handling of objects if excessive number of objects identified: Continue
Maximum number of objects: 500
Use advanced settings?: Yes
Threshold setting version: 12
Threshold strategy:Global
Thresholding method:Otsu
Threshold smoothing scale: 1.3488
Threshold correction factor:0.2
Lower and upper bounds on threshold: 0; 1
Manual threshold:0.0
Select the measurement to threshold with: None
Two-class or three-class thresholding?: Two classes
Log transform before thresholding?: No
Assign pixels in the middle intensity class to the foreground or the background?: Foreground
Size of adaptive window:20
Lower outlier fraction:0.05
Upper outlier fraction:0.05
Averaging method:Mean
Variance method:Standard deviation
# of deviations:1
Thresholding method:Otsu
IdentifyPrimaryObjects:[module_num:10|svn_version:'Unknown'|variable_revision_number:15|show_window:False|notes:[]|batch_state:array([] dtype=uint8)|enabled: True|wants_pause:False]
Select the input image: EnhancedGreen
Name the primary objects to be identified: Cilia
Typical diameter of objects in pixel units (Min, Max): 10- 220
Discard objects outside the diameter range?:Yes
Discard objects touching the border of the image?:Yes
Method to distinguish clumped objects: Intensity
Method to draw dividing lines between clumped objects: Propagate
Size of smoothing filter:100
Suppress local maxima that are closer than this minimum allowed distance:400
Speed up by using lower-resolution image to find local maxima?:Yes
Fill holes in identified objects?: After both thresholding and declumping
Automatically calculate size of smoothing filter for declumping?: No
Automatically calculate minimum allowed distance between local maxima?:No
Handling of objects if excessive number of objects identified: Continue
Maximum number of objects:500
Use advanced settings?:Yes
Threshold setting version:12
Threshold strategy:Global
Thresholding method:Otsu
Threshold smoothing scale:0.2
Threshold correction factor:0.8
Lower and upper bounds on threshold: 0.0; 1.0

Manual threshold:0.0
Select the measurement to threshold with: None
Two-class or three-class thresholding?:Three classes
Log transform before thresholding?:No
Assign pixels in the middle intensity class to the foreground or the background?:Foreground
Size of adaptive window:50
Lower outlier fraction:0.05
Upper outlier fraction:0.05
Averaging method:Mean
Variance method:Standard deviation
# of deviations:2.0
Thresholding method:Otsu
MeasureObjectSizeShape:[module_num:11|svn_version:'Unknown'|variable_revision_number:3|show_window:False|notes:[]|batch_state:array([] dtype=uint8)|enabled: True|wants_pause:False]
Select object sets to measure: Cilia
Calculate the Zernike features?:No
Calculate the advanced features?:No
DisplayDataOnImage:[module_num:12|svn_version:'Unknown'|variable_revision_number:6|show_window:False|notes:[]|batch_state:array([] dtype=uint8)|enabled:True|wants_pause:False]
Display object or image measurements?:Object
Select the input objects: Cilia
Measurement to display: AreaShape_MajorAxisLength
Select the image on which to display the measurements: EnhancedGreen
Text color:#5BFF5B
Name the output image that has the measurements displayed: DisplayImage
Font size (points):9
Number of decimals:1
Image elements to save: Image
Annotation offset (in pixels):10
Display mode:Text
Color map:Default
Display background image?:Yes
Color map scale:Use this image's measurement range
Color map range: 0.0; 1.0
Font:Arial
Use scientific notation?:No
Font weight:normal
FilterObjects:[module_num:13|svn_version:'Unknown'|variable_revision_number:10|show_window:False|notes:[]|batch_state:array([] dtype=uint8)|enabled: True|wants_pause:False]
Select the objects to filter: Cilia
Name the output objects: FilterdCilia
Select the filtering mode: Measurements
Select the filtering method: Limits
Select the objects that contain the filtered objects: None
Select the location of the rules or classifier file: Elsewhere..
Rules or classifier file name: rules.txt
Class number:1
Measurement count:1
Additional object count:0
Assign overlapping child to: Both parents
Keep removed objects as a separate set?: Yes
Name the objects removed by the filter: RemovedCilia
Select the measurement to filter by: AreaShape_MajorAxisLength

Filter using a minimum measurement value?: Yes; Minimum value:18
Filter using a maximum measurement value?: No; Maximum value:400
Allow fuzzy feature matching?:No
MeasureObjectSizeShape:[module_num:14|svn_version:'Unknown'|variable_revision_number:3|show_window:False|notes:[]|batch_state:array([] dtype=uint8)|enabled:True|wants_pause:False]
Select object sets to measure: FilterdCilia
Calculate the Zernike features?:No
Calculate the advanced features?:No
OverlayOutlines:[module_num:15|svn_version:'Unknown'|variable_revision_number:4|show_window:False|notes:[]|batch_state:array([] dtype=uint8)|enabled: True|wants_pause:False]
Display outlines on a blank image?:No
Select image on which to display outlines: original
Name the output image: Overlayed_Nuclei_Cilia
Outline display mode:Color
Select method to determine brightness of outlines: Max of image
How to outline:Thick
Select outline color:#00CCCC
Select objects to display: Nuclei
Select outline color:yellow
Select objects to display: FilterdCilia
Select outline color:red
Select objects to display: RemovedCilia
OverlayOutlines:[module_num:16|svn_version:'Unknown'|variable_revision_number:4|show_window:False|notes:[]|batch_state:array([] dtype=uint8)|enabled: True|wants_pause: False]
Display outlines on a blank image?:No
Select image on which to display outlines: original
Name the output image: Overlayed_Cilia
Outline display mode:Color
Select method to determine brightness of outlines: Max of image
How to outline:Thick
Select outline color:yellow
Select objects to display: FilterdCilia
DisplayDataOnImage:[module_num:17|svn_version:'Unknown'|variable_revision_number:6|show_window:False|notes:[]|batch_state:array([] dtype=uint8)|enabled: True|wants_pause:False]
Display object or image measurements?:Object
Select the input objects: FilterdCilia
Measurement to display: AreaShape_MajorAxisLength
Select the image on which to display the measurements: Overlayed_Nuclei_Cilia
Text color:white
Name the output image that has the measurements displayed: DisplayCilia_Lenght
Font size (points):15
Number of decimals:1
Image elements to save: Image
Annotation offset (in pixels):40
Display mode:Text
Color map:Default
Display background image?:Yes
Color map scale: Use this image's measurement range 1.0
Color map range:0.0
Font:Arial
Use scientific notation?:No
Font weight:bold

DisplayDataOnImage:[module_num:18|svn_version:'Unknown'|variable_revision_number:6|show_window:False|notes:[]|batch_state:array([] dtype=uint8)|enabled: True|wants_pause:False]
Display object or image measurements?:Object
Select the input objects: FilterdCilia
Measurement to display: AreaShape_MajorAxisLength
Select the image on which to display the measurements: Overlayed_Cilia
Text color:white
Name the output image that has the measurements displayed: DisplayCilia_Lenght_wIo_nuc
Font size (points):15
Number of decimals:1
Image elements to save: Image
Annotation offset (in pixels):40
Display mode:Text
Color map:Default
Display background image?:Yes
Color map scale: Use this image's measurement range
Color map range: 0.0; 1.0
Font:Arial
Use scientific notation?:No
Font weight:bold
SaveImages:[module_num:19|svn_version:'Unknown'|variable_revision_number:16|show_window:False|notes:['Save the color image as an 8-bit TIF appending the text RBG to the original filename of the DAPI image.']|batch_state:array([]
Select the type of image to save: Image
Select the image to save: DisplayCilia_Lenght
Select method for constructing file names: From image filename
Select image name for file prefix: original
Enter single file name: CroppedFlyImage
Number of digits:4
Append a suffix to the image file name?:Yes
Text to append to the image name: overlay
Saved file format:tiff
Output file location: Elsewhere...|C:\\\\Users\\\\mrehders\\\\Desktop\\\\out
Image bit depth:8-bit integer
Overwrite existing files without warning?: No
When to save: Every cycle
Record the file and path information to the saved image?:No
Create subfolders in the output folder?:No
Base image folder:Default Input Folder
How to save the series:T (Time)
Save with lossless compression?:No
SaveImages:[module_num:20|svn_version:'Unknown'|variable_revision_number:16|show_window:False|notes:[]|batch_state:array([] dtype=uint8)|enabled: True|wants_pause:False]
Select the type of image to save: Image
Select the image to save: DisplayCilia_Lenght_wIo_nuc
Select method for constructing file names: From image filename
Select image name for file prefix: original
Enter single file name: OrigBlue
Number of digits:4
Append a suffix to the image file name?:Yes
Text to append to the image name: cilia_only
Saved file format:tiff
Output file location: Elsewhere...|C:\\\\Users\\\\mrehders\\\\Desktop\\\\out

Image bit depth:8-bit integer
Overwrite existing files without warning?:No
When to save: Every cycle
Record the file and path information to the saved image?:No
Create subfolders in the output folder?:No
Base image folder:Elsewhere...|
How to save the series:T (Time)
Save with lossless compression?:Yes
ExportToSpreadsheet:[module_num:21|svn_version:'Unknown'|variable_revision_number:13|show_window:False|notes:[""Export any measurements to a comma-delimited file (.csv). The measurements made for the nuclei cell and cytoplasm objects will be saved to separate .csv files in addition to the per-image .csv's.""]|batch_state:array([]
Select the column delimiter: Comma ("")
Add image metadata columns to your object data file?:No
Add image file and folder names to your object data file?:No
Select the measurements to export: Yes
Calculate the per-image mean values for object measurements?:Yes
Calculate the per-image median values for object measurements?:No
Calculate the per-image standard deviation values for object measurements?: No
Output file location: Elsewhere...|C:\\Users\\mrehders\\Desktop\\out
Create a GenePattern GCT file?:No
Select source of sample row name: Metadata
Select the image to use as the identifier: None
Select the metadata to use as the identifier: None
Export all measurement types?:No
Press button to select measurements: FilterdCilia|AreaShape_BoundingBoxArea
FilterdCilia|AreaShape_BoundingBoxMaximum_X
FilterdCilia|AreaShape_BoundingBoxMaximum_Y
FilterdCilia|AreaShape_Area
FilterdCilia|AreaShape_BoundingBoxMinimum_X
FilterdCilia|AreaShape_BoundingBoxMinimum_Y
FilterdCilia|AreaShape_Eccentricity
FilterdCilia|AreaShape_MinorAxisLength
FilterdCilia|AreaShape_MajorAxisLength
FilterdCilia|AreaShape_Perimeter
FilterdCilia|AreaShape_EulerNumber
FilterdCilia|AreaShape_FormFactor
FilterdCilia|AreaShape_Extent
Image|Count_Nuclei
Image|Count_FilterdCilia
Image|Count_Cilia
Image|FileName_original
Experiment|Pipeline_Pipeline
Representation of Nan/Inf: NaN
Add a prefix to file names?:No
Filename prefix:MyExpt_
Overwrite existing files without warning?:Yes
Data to export:Image
Combine these object measurements with those of the previous object?: No
File name:Image.csv
Use the object name for the file name?:No
Data to export:FilterdCilia
Combine these object measurements with those of the previous object?:No

File name:CiliaFiltered.csv
Use the object name for the file name?:No
Data to export:Experiment
Combine these object measurements with those of the previous object?:No
File name:DATA.csv
Use the object name for the file name?:Yes

Supplementary Table 2: CellProfiler™ Pipeline Settings “non-expert user”.

<table>
<tr><td>CellProfiler_Version,4.2.6</td></tr>
<tr><td>ChannelType_OrigColor,Color</td></tr>
<tr><td>Pipeline_Pipeline,"CellProfiler Pipeline: http://www.cellprofiler.org</td></tr>
<tr><td>Version:5</td></tr>
<tr><td>DateRevision:426</td></tr>
<tr><td>ModuleCount:17</td></tr>
<tr><td>HasImagePlaneDetails:False</td></tr>
<tr><td>Images:[module_num:1|svn_version:'Unknown'|variable_revision_number:2|show_window:False|notes:['To begin creating your project, use the Images module to compile a list of files and/or folders that you want to analyze. You can also specify a set of rules to include only the desired files in your selected folders.']|batch_state:array([], dtype=uint8)|enabled:True|wants_pause:False]</td></tr>
<tr><td>Filter images?:Images only</td></tr>
<tr><td>Select the rule criteria:and (extension does isimage) (directory doesnot containregexp ""[\\\\/]\\."")</td></tr>
<tr><td>Metadata:[module_num:2|svn_version:'Unknown'|variable_revision_number:6|show_window:False|notes:['The Metadata module optionally allows you to extract information describing your images (i.e, metadata) which will be stored along with your measurements. This information can be contained in the file name and/or location, or in an external file.']|batch_state:array([], dtype=uint8)|enabled:True|wants_pause:False]</td></tr>
<tr><td>Extract metadata?:No</td></tr>
<tr><td>Metadata data type:Text</td></tr>
<tr><td>Metadata types:{}</td></tr>
<tr><td>Extraction method count:1</td></tr>
<tr><td>Metadata extraction method:Extract from file/folder names</td></tr>
<tr><td>Metadata source:File name</td></tr>
<tr><td>Regular expression to extract from file name:^(?P<Plate>.*)_(?P<Well>[A-P][0-9]{2})_s(?P<Site>[0-9])_w(?P<ChannelNumber>[0-9])</td></tr>
<tr><td>Regular expression to extract from folder name:(?P<Date>[0-9]{4}_[0-9]{2}_[0-9]{2})$</td></tr>
<tr><td>Extract metadata from:All images</td></tr>
<tr><td>Select the filtering criteria:and (file does contain """")</td></tr>
<tr><td>Metadata file location:Elsewhere...|</td></tr>
<tr><td>Match file and image metadata:[]</td></tr>
<tr><td>Use case insensitive matching?:No</td></tr>
<tr><td>Metadata file name:None</td></tr>
<tr><td>Does cached metadata exist?:No</td></tr>
<tr><td>NamesAndTypes:[module_num:3|svn_version:'Unknown'|variable_revision_number:8|show_window:False|notes:['The NamesAndTypes module allows you to assign a meaningful name to each image by which other modules will refer to it.']|batch_state:array([], dtype=uint8)|enabled:True|wants_pause:False]</td></tr>
<tr><td>Assign a name to:All images</td></tr>
<tr><td>Select the image type: Color image</td></tr>
<tr><td>Name to assign these images: OrigColor</td></tr>
<tr><td>Match metadata:[]</td></tr>
<tr><td>Image set matching method: Order</td></tr>
<tr><td>Set intensity range from: Image metadata</td></tr>
<tr><td>Assignments count:1</td></tr>
<tr><td>Single images count:0</td></tr>
<tr><td>Maximum intensity:255.0</td></tr>
<tr><td>Process as 3D?:No</td></tr>
<tr><td>Relative pixel spacing in X:1.0</td></tr>
<tr><td>Relative pixel spacing in Y:1.0</td></tr>
<tr><td>Relative pixel spacing in Z:1.0</td></tr>
<tr><td>Select the rule criteria: and (file does contain ""nuclei"")</td></tr>
<tr><td>Name to assign these images: Nuc</td></tr>
</table>

Name to assign these objects: Cell
Select the image type: Grayscale image
Set intensity range from: Image metadata
Maximum intensity:255.0
Groups:[module_num:4|svn_version:'Unknown'|variable_revision_number:2|show_window:False|notes:['The Groups module optionally allows you to split your list of images into image subsets (groups) which will be processed independently of each other. Examples of groupings include screening batches, microtiter plates, time-lapse movies, etc.']|batch_state:array([], dtype=uint8)|enabled:True|wants_pause:False]
Do you want to group your images?:No
grouping metadata count:1
Metadata category:None
ColorToGray:[module_num:5|svn_version:'Unknown'|variable_revision_number:4|show_window:False|notes:[]|batch_state:array([], dtype=uint8)|enabled:True|wants_pause:False]
Select the input image: OrigColor
Conversion method:Split
Image type:RGB
Name the output image:OrigGray
Relative weight of the red channel:1.0
Relative weight of the green channel:1.0
Relative weight of the blue channel:1.0
Convert red to gray?:Yes
Name the output image: OrigRed
Convert green to gray?:Yes
Name the output image: OrigGreen
Convert blue to gray?:Yes
Name the output image: OrigBlue
Convert hue to gray?:Yes
Name the output image: OrigHue
Convert saturation to gray?:Yes
Name the output image: OrigSaturation
Convert value to gray?:Yes
Name the output image: OrigValue
Channel count:1
Channel number:1
Relative weight of the channel:1.0
Image name:Channel1
IdentifyPrimaryObjects:[module_num:6|svn_version:'Unknown'|variable_revision_number:15|show_window:False|notes:[]|batch_state:array([], dtype=uint8)|enabled:True|wants_pause:False]
Select the input image:OrigBlue
Name the primary objects to be identified: NucleiCount
Typical diameter of objects, in pixel units (Min,Max):245,500
Discard objects outside the diameter range?:Yes
Discard objects touching the border of the image?:No
Method to distinguish clumped objects: Intensity
Method to draw dividing lines between clumped objects: Propagate
Size of smoothing filter:60
Suppress local maxima that are closer than this minimum allowed distance:136.7
Speed up by using lower-resolution image to find local maxima?:No
Fill holes in identified objects?:After both thresholding and declumping
Automatically calculate size of smoothing filter for declumping?:No
Automatically calculate minimum allowed distance between local maxima?:No
Handling of objects if excessive number of objects identified: Continue
Maximum number of objects:500
Use advanced settings?:Yes

Threshold setting version:12
Threshold strategy:Global
Thresholding method: Minimum Cross-Entropy
Threshold smoothing scale:1.3488
Threshold correction factor:1.5
Lower and upper bounds on threshold:0.1,1
Manual threshold:0.0
Select the measurement to threshold with: None
Two-class or three-class thresholding?: Two classes
Log transform before thresholding?: No
Assign pixels in the middle intensity class to the foreground or the background?: Foreground
Size of adaptive window:50
Lower outlier fraction:0.05
Upper outlier fraction:0.05
Averaging method:Mean
Variance method:Standard deviation
# of deviations:1.0
Thresholding method: Minimum Cross-Entropy
MeasureImageIntensity:[module_num:7|svn_version:'Unknown'|variable_revision_number:4|show_window:False|notes:[]|batch_state:array([], dtype=uint8)|enabled: True|wants_pause:False]
Select images to measure: OrigRed
Measure the intensity only from areas enclosed by objects?:No
Select input object sets:
Calculate custom percentiles:No
Specify percentiles to measure:10,90
IdentifyPrimaryObjects:[module_num:8|svn_version:'Unknown'|variable_revision_number:15|show_window:False|notes:[]|batch_state:array([], dtype=uint8)|enabled: True|wants_pause:False]
Select the input image: OrigGreen
Name the primary objects to be identified: CiliaCount
Typical diameter of objects, in pixel units (Min,Max):15,90
Discard objects outside the diameter range?:Yes
Discard objects touching the border of the image?:Yes
Method to distinguish clumped objects: Shape
Method to draw dividing lines between clumped objects: Propagate
Size of smoothing filter:1
Suppress local maxima that are closer than this minimum allowed distance:150
Speed up by using lower-resolution image to find local maxima?:Yes
Fill holes in identified objects?:After both thresholding and declumping
Automatically calculate size of smoothing filter for declumping?:No
Automatically calculate minimum allowed distance between local maxima?:No
Handling of objects if excessive number of objects identified: Continue
Maximum number of objects:500
Use advanced settings?:Yes
Threshold setting version:12
Threshold strategy:Global
Thresholding method:Robust Background
Threshold smoothing scale:1.3488
Threshold correction factor:1.0
Lower and upper bounds on threshold:0.1,1.0
Manual threshold:0.0
Select the measurement to threshold with: None
Two-class or three-class thresholding?:Two classes

Log transform before thresholding?:No
Assign pixels in the middle intensity class to the foreground or the background?:Foreground
Size of adaptive window: 50
Lower outlier fraction: 0.1
Upper outlier fraction: 0.05
Averaging method:Mean
Variance method:Standard deviation
# of deviations:10.0
Thresholding method: Minimum Cross-Entropy
MeasureObjectSizeShape:[module_num:9|svn_version:'Unknown'|variable_revision_number:3|show_window:False|notes:[]|batch_state:array([], dtype=uint8)|enabled:True|wants_pause:False]
Select object sets to measure: CiliaCount
Calculate the Zernike features?:No
Calculate the advanced features?:No
ExportToSpreadsheet:[module_num:10|svn_version:'Unknown'|variable_revision_number:13|show_window:False|notes:[]|batch_state:array([], dtype=uint8)|enabled:True|wants_pause:False]
Select the column delimiter: Comma ("","")
Add image metadata columns to your object data file?:No
Add image file and folder names to your object data file?:No
Select the measurements to export: Yes
Calculate the per-image mean values for object measurements?:No
Calculate the per-image median values for object measurements?:No
Calculate the per-image standard deviation values for object measurements?:No
Output file location:Elsewhere...|C:\\Users\\mrehders\\Desktop\\Output_Inhib_1h_CP_Joao
Create a GenePattern GCT file?:No
Select source of sample row name: Metadata
Select the image to use as the identifier: None
Select the metadata to use as the identifier: None
Export all measurement types?:Yes
Press button to select measurements:Image|FileName_OrigColor,Image|Intensity_TotalIntensity_OrigRed,Image|Count_NucleiCount,CiliaCount|AreaShape_Area,CiliaCount|AreaShape_MajorAxisLength,CiliaCount|AreaShape_Eccentricity,CiliaCount|AreaShape_FormFactor,CiliaCount|AreaShape_Perimeter,CiliaCount|Number_Object_Number
Representation of Nan/Inf:NaN
Add a prefix to file names?:Yes
Filename prefix:BandL/BandL
Overwrite existing files without warning?:No
Data to export:Image
Combine these object measurements with those of the previous object?:No
File name:DATA.csv
Use the object name for the file name?:Yes
OverlayOutlines:[module_num:11|svn_version:'Unknown'|variable_revision_number:4|show_window:False|notes:[]|batch_state:array([], dtype=uint8)|enabled:True|wants_pause:False]
Display outlines on a blank image?:No
Select image on which to display outlines: OrigBlue
Name the output image: BlueOverlay
Outline display mode:Color
Select method to determine brightness of outlines: Max of image
How to outline:Thick
Select outline color:#FF00E9
Select objects to display: NucleiCount
OverlayOutlines:[module_num:12|svn_version:'Unknown'|variable_revision_number:4|show_window:False|notes:[]|batch_state:array([], dtype=uint8)|enabled:True|wants_pause:False]
Display outlines on a blank image?:No

Select image on which to display outlines: OrigColor
Name the output image: OverlayNuclei
Outline display mode:Color
Select method to determine brightness of outlines: Max of image
How to outline:Thick
Select outline color:#FF00E4
Select objects to display: NucleiCount
OverlayOutlines:[module_num:13|svn_version:'Unknown'|variable_revision_number:4|show_window:False|notes:[]|batch_state:array([], dtype=uint8)|enabled:True|wants_pause:False]
Display outlines on a blank image?:No
Select image on which to display outlines: OrigGreen
Name the output image: GreenOverlay
Outline display mode:Color
Select method to determine brightness of outlines: Max of image
How to outline:Thick
Select outline color:#00F200
Select objects to display: CiliaCount
DisplayDataOnImage:[module_num:14|svn_version:'Unknown'|variable_revision_number:6|show_window:False|notes:[]|batch_state:array([], dtype=uint8)|enabled:True|wants_pause:False]
Display object or image measurements?:Object
Select the input objects: CiliaCount
Measurement to display: AreaShape_MajorAxisLength
Select the image on which to display the measurements: GreenOverlay
Text color:#FF00EC
Name the output image that has the measurements displayed :DisplayCilia
Font size (points):20
Number of decimals:1
Image elements to save: Image
Annotation offset (in pixels):0
Display mode:Text
Color map:Default
Display background image?:Yes
Color map scale:Use this image's measurement range
Color map range:0.0,1.0
Use scientific notation?:No
Font weight:normal
SaveImages:[module_num:15|svn_version:'Unknown'|variable_revision_number:16|show_window:False|notes:[]|batch_state:array([], dtype=uint8)|enabled: True|wants_pause: False]
Select the type of image to save: Image
Select the image to save: DisplayCilia
Select method for constructing file names: From image filename
Select image name for file prefix: OrigColor
Enter single file name: OrigBlue
Number of digits:4
Append a suffix to the image file name?:Yes
Text to append to the image name: Green
Saved file format:tiff
Output file location:Elsewhere...|C:\\Users\\mrehders\\Desktop\\Output_Inhib_1h_CP_Joao
Image bit depth:8-bit integer
Overwrite existing files without warning?:No
When to save:Every cycle
Record the file and path information to the saved image?:No

Create subfolders in the output folder?:No
Base image folder:Elsewhere...|
How to save the series:T (Time)
Save with lossless compression?:Yes
SaveImages:[module_num:16|svn_version:'Unknown'|variable_revision_number:16|show_window:False|notes:[]|batch_state:array([], dtype=uint8)|enabled:True|wants_pause:False]
Select the type of image to save: Image
Select the image to save: BlueOverlay
Select method for constructing file names: From image filename
Select image name for file prefix: OrigColor
Enter single file name:OrigBlue
Number of digits:4
Append a suffix to the image file name?:Yes
Text to append to the image name:Pink1
Saved file format:tiff
Output file location:Elsewhere...|C:\\\\Users\\\\mrehders\\\\Desktop\\\\Output_Inhib_1h_CP_Joao
Image bit depth:8-bit integer
Overwrite existing files without warning?:No
When to save: Every cycle
Record the file and path information to the saved image?:No
Create subfolders in the output folder?:No
Base image folder:Elsewhere...|
How to save the series:T (Time)
Save with lossless compression?:Yes
SaveImages:[module_num:17|svn_version:'Unknown'|variable_revision_number:16|show_window:False|notes:[]|batch_state:array([], dtype=uint8)|enabled:True|wants_pause:False]
Select the type of image to save: Image
Select the image to save: OverlayNuclei
Select method for constructing file names: From image filename
Select image name for file prefix: OrigColor
Enter single file name: OrigBlue
Number of digits:4
Append a suffix to the image file name?:Yes
Text to append to the image name: PinkAll
Saved file format:tiff
Output file location:Elsewhere...|C:\\\\Users\\\\mrehders\\\\Desktop\\\\Output_Inhib_1h_CP_Joao
Image bit depth:8-bit integer
Overwrite existing files without warning?:No
When to save: Every cycle
Record the file and path information to the saved image?:No
Create subfolders in the output folder?:No
Base image folder:Elsewhere...|
How to save the series:T (Time)
Save with lossless compression?:Yes

Supplementary Table 3: Machine Learning Terminology. Since the proposed cell segmentation and cilia detection approaches are based on ML, in this section we introduce the terms necessary to describe a segmentation and detection ML model and to interpret the results using it.

**Ground truth**	Expert-annotated data — information that is known to be real or true, provided by direct observation and measurement. This information is presented in the form of labels produced by human expert-annotators.
**Neural network fine-tuning**	ML technique in which the weights (main parameters) of a pre-trained model are trained on new data. This is one of the key concepts in neural network training.
**Few-shot fine-tuning**	Train datasets in ML usually are very big (hundreds and thousands of labeled images). Few-shot fine-tuning is the ability of the ML model to be fine-tuned with good performance on the test set using a relatively small number of data elements (only few labeled images).
**Support set images (SIS)**	In the few-shot setting, it is the data element (image) that is used for model training. The number of support set images (SIN) is much less than the number of all images in the train set. In the few-shot setting of **CU Cilia**, we used support set images from each experiment that was included in the train set.
**Model hyperparameters**	Set of parameters that are crucial for ML model performance (learning rate, batch size, weight decay (ML technique to prevent overfitting on train set), etc.).
**K-fold cross-validation**	Procedure used to estimate the performance of the model on new data. During the k-fold cross-validation procedure, the dataset is split into a k-number of folds and is used to evaluate the model's ability when given new data. K refers to the number of groups the data is split into.
**Intersection over union (IoU)**	It is a number that quantifies the degree of overlap between two boxes. In the case of object detection and segmentation, IoU evaluates the overlap of the Ground Truth and Prediction region. High IoU leads to more strict and precise results.

Supplementary Table 4: List of optimal hyperparameters.

	**Segmentation Model type A**	**Segmentation Model type A + B**
Learning rate	0.05358	0.0397
Weight decay	0.001215	0.00001254
Batch size	4	8
Number of epochs	30	70
Number of images per epoch	45	20

Supplementary Table 5: List of optimal hyperparameters for the few-shot setting.

	**Segmentation Model type A (SIN = 5)**	**Segmentation Model type A + B (SIN = 5)**
Learning rate	0.0248	0.01237
Weight decay	0.00001	0.0005402
Batch size	8	8
Number of epochs	30	70
Number of images per epoch	35	5

Supplementary Table 6: Comparison of experiments based on average metrics obtained over "Nthy-ori_DCG04_E64" test subset. The table displays the average values with standard deviation after 50 test launches of the corresponding experiment.

**Segmentation model experiment type**	**AP at 0.5 IoU (high is better)**	**AP at 0.75 IoU (high is better)**	**Mean |1 - CNR| (lower is better)**
Cellpose	0.797	0.618	0.031
Model A	0.857 ± 0.004	0.692 ± 0.006	0.041 ± 0.005
Model A + B	0.896 ± 0.002	0.798 ± 0.002	**0.014 ± 0.002**
Model A (SIN = 5)	**0.897 ± 0.002**	0.819 ± 0.004	0.016 ± 0.002
Model A + B (SIN = 5)	**0.897 ± 0.002**	**0.819 ± 0.003**	0.016 ± 0.003

Supplementary Table 7: Comparison of experiments based on average metrics obtained over "Nthy-ori_E64" test subset. The table displays the average values with standard deviation after 50 test launches of the corresponding experiment.

**Segmentation model experiment type**	**AP at 0.5 IoU (high is better)**	**AP at 0.75 IoU (high is better)**	**Mean |1 - CNR| (lower is better)**
Cellpose	0.744	0.624	**0.006**
Model A	**0.817 ± 0.006**	**0.686 ± 0.006**	0.025 ± 0.005
Model A + B	0.785 ± 0.003	0.663 ± 0.002	0.049 ± 0.003
Model A (SIN = 5)	0.781 ± 0.003	0.649 ± 0.001	0.024 ± 0.003
Model A + B (SIS = 5)	0.781 ± 0.003	0.649 ± 0.002	0.024 ± 0.004

Supplementary Table 8: Comparison of experiments based on average metrics obtained over "Nthy-ori_E64d" test subset. The table displays the average values with standard deviation after 50 test launches of the corresponding experiment.

**Segmentation model experiment type**	AP at 0.5 IoU (high is better)	**AP at 0.75 IoU (high is better)**	**Mean |1 - CNR| (lower is better)**
Cellpose	0.804	0.666	0.039
Model A	0.851 ± 0.005	**0.726 ± 0.005**	0.004 ± 0.003
Model A + B	**0.859 ± 0.002**	0.714 ± 0.001	**0.044 ± 0.002**
Model A (SIN = 5)	0.854 ± 0.002	0.699 ± 0.005	0.051 ± 0.005
Model A + B (SIN = 5)	0.854 ± 0.003	0.699 ± 0.007	0.051 ± 0.004

Supplementary Table 9: Comparison of experiments based on average metrics obtained over "Nthy-ori_TSH" test subset. The table displays the average values with standard deviation after 50 test launches of the corresponding experiment.

**Segmentation model experiment type**	**AP at 0.5 IoU (high is better)**	**AP at 0.75 IoU (high is better)**	**Mean |1 - CNR| (lower is better)**
Cellpose	**0.855**	0.714	0.020
Model A	0.850 ± 0.005	**0.723 ± 0.006**	**0.004 ± 0.003**
Model A + B	0.813 ± 0.002	0.640 ± 0.002	0.046 ± 0.003
Model A (SIN = 5)	0.821 ± 0.003	0.642 ± 0.007	0.046 ± 0.007
Model A + B (SIN = 5)	0.821 ± 0.003	0.642 ± 0.006	0.047 ± 0.004
